# A New Family of Iron(II)-Cyclopentadienyl Compounds Shows Strong Activity against Colorectal and Triple Negative Breast Cancer Cells

**DOI:** 10.3390/molecules25071592

**Published:** 2020-03-30

**Authors:** Adhan Pilon, Ana Rita Brás, Leonor Côrte-Real, Fernando Avecilla, Paulo J. Costa, Ana Preto, M. Helena Garcia, Andreia Valente

**Affiliations:** 1 Centro de Química Estrutural, Faculdade de Ciências, Universidade de Lisboa, Campo Grande, 1749-016 Lisboa, Portugal; fc42762@alunos.fc.ul.pt (A.P.); pg31015@alunos.uminho.pt (A.R.B.); ldcortereal@fc.ul.pt (L.C.-R.); 2 Departamento de Química e Bioquímica, Faculdade de Ciências, Universidade de Lisboa, Campo Grande, 1749-016 Lisboa, Portugal; 3 Centre of Molecular and Environmental Biology, Department of Biology, University of Minho, Campus de Gualtar, 4710-057 Braga, Portugal; apreto@bio.uminho.pt; 4 Institute of Science and Innovation for Bio-Sustainability, University of Minho, Campus de Gualtar, Edifício 18, 4710-057 Braga, Portugal; 5 Grupo Xenomar, Centro de Investigacións Científicas Avanzadas (CICA), Departamento de Química, Facultade de Ciencias, Universidade da Coruña, Campus de A Coruña, 15071 A Coruña, Spain; fernando.avecilla@udc.es; 6 Faculty of Sciences, University of Lisboa, BioISI—Biosystems & Integrative Sciences Institute, Campo Grande, C8 bdg, 1749-016 Lisboa, Portugal; pjcosta@fc.ul.pt

**Keywords:** iron(II)-cyclopentadienyl, nitrile-based ligands, colorectal cancer, triple negative breast cancer

## Abstract

A family of compounds with the general formula [Fe(η^5^-C_5_H_5_)(CO)(PPh_3_)(NCR)]^+^ has been synthesized (NCR = benzonitrile (**1**); 4-hydroxybenzonitrile (**2**); 4-hydroxymethylbenzonitrile (**3**); 4-aminobenzonitrile (**4**); 4-bromobenzonitrile (**5**); and, 4-chlorocinnamonitrile (**6**)). All of the compounds were obtained in good yields and were completely characterized by standard spectroscopic and analytical techniques. Compounds **1**, **4**, and **5** crystallize in the monoclinc P21/c space group and packing is determined by short contacts between the phosphane phenyl rings and cyclopentadienyl (compounds **1** and **4**) or π-π lateral interactions between the benzonitrile molecules (complex **5**). DFT and TD-DFT calculations were performed to help in the interpretation of the experimental UV-Vis. data and assign the electronic transitions. Cytotoxicity studies in MDA-MB-231 breast and SW480 colorectal cancer-derived cell lines showed IC_50_ values at a low micromolar range for all of the compounds in both cell lines. The determination of the selectivity index for colorectal cells (SW480 vs. NCM460, a normal colon-derived cell line) indicates that the compounds have some inherent selectivity. Further studies on the SW480 cell line demonstrated that the compounds induce cell death by apoptosis, inhibit proliferation by inhibiting the formation of colonies, and affect the actin-cytoskeleton of the cells. These results are not observed for the hydroxylated compounds **2** and **3**, where an alternative mode of action might be present. Overall, the results indicate that the substituent at the nitrile-based ligand is associated to the biological activity of the compounds.

## 1. Introduction

**Figure 1:**
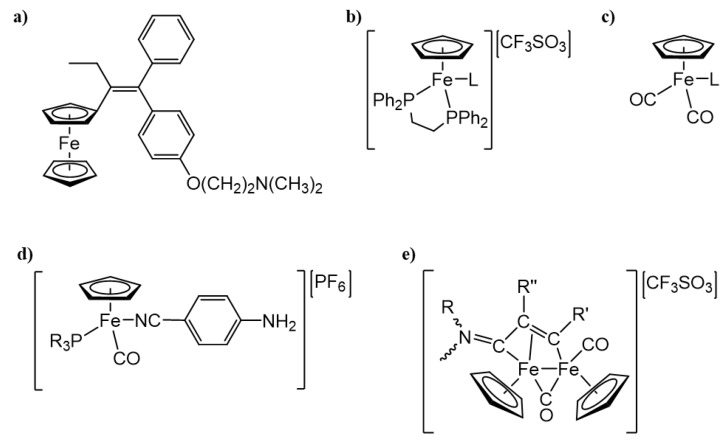
Organometallic iron-based compounds with potential as anticancer agents. (**a**) Ferrocifen; (**b**) L = imidazole or nitrile based ligand; (**c**) L = Cl, Br, I, isothiocyanates; (**d**) PR_3_ = phosphane-based ligand; and, (**e**) R = Me, xyl, CH_2_Ph; R′ = Ph, 2-naphthyl, 3-C_6_H_4_OH, 4-C_6_H_4_CO_2_H, 3-thiophenyl, Me, CO_2_Me, 2-pyridyl; R″ = H, CO_2_Me, Me, Ph.

Cancer is among one of the deadliest diseases worldwide. Most of the chemotherapeutic agents that are used to treat this illness are organic molecules, yet, the success of transition metal complexes for cancer treatments is well demonstrated by the clinical use of platinum-based agents and the presence of several metal-based compounds in clinical trials [1]. Despite such successes, the lack of selectivity and multidrug resistance of such metallodrugs constitutes a serious challenge to overcome. In that frame, the use of biologically essential metals, such as iron, seems to be a valuable strategy [2]. The better-known example is the ferrocifen family of compounds, ferrocenyl analogues of tamoxifen, developed by Jaouen and coworkers [2,3,4,5,6,7,8]. Tamoxifen is the first line chemotherapeutic for patients with hormone-dependent breast cancer (oestrogen receptor α-positive, ERα+). The replacement of a phenyl group in tamoxifen by a ferrocenyl group originated compounds that are highly active against both ERα+ and hormone-independent (ERα−) breast cancer cells (Figure 1a). Although this was an outstanding result, the ferrocifen-based compounds suffer from poor bioavailability, considerably delaying their entrance into clinical trials. Several formulations are being tested in order to overcome this limitation, such as nanoparticles or lipid nanocapsules [5,9].

We have been exploring the use of half-sandwich ruthenium(II)-cyclopentadienyl-based compounds as prospective anticancer agents [10]. Recently, we have successfully dedicated our studies to cancers without clinical cure, such as triple negative breast cancer (TNBC) [11,12,13,14,15,16] and colorectal cancer (CRC) with KRAS or BRAF mutations [17]. Given the promising results that were obtained with ruthenium, and since iron belongs to the same Group of the Periodic Table, being more abundant and cheaper, the exploitation of iron(II)-cyclopentadienyl-based compounds merits being explored. Our preliminary results on the [Fe(η^5^-Cp)(dppe)(L)]^+^ compounds, where L is an imidazole [18], a quinolinecarbonitrile [19], or a pyrazinecarbonitrile [19]-based ligand (Figure 1b), have validated the strategy. Later works by us [20], Fan et al., [21], and Dyson, Marchetti et al., [22] on {Fe(Cp)(CO)_n_} or {Fe_2_(Cp)_2_(CO)_2_} based-compounds showed that the replacement of dppe by monodentate ligands allows for the synthesis of stable and more water soluble complexes (Figure 1c–e). In particular, we have explored different substituents on the phosphane ligands of [Fe(η^5^-Cp)(CO)(phosphane)(aminobenzonitrile)]^+^ compounds, concluding that triphenylphosphane was the ligand that imparted a better cytotoxic profile to the compounds on human cervical cancer cells (HeLa) [20]. On the continuation of this structure-activity work, our strategy of molecular design has now included structural changes on the nitrile-based ligand. Thus, a series of five benzonitriles with different substituents at the *para* position (H, OH, CH_2_OH, NH_2_, and Br) were used, as well as a longer aromatic system (4-chlorocinnamonitrile). The influence of the nitriles on the complexes’ activity was assessed in two different cancer cell lines (breast and colorectal). The results revealed that the activity of the compounds is dependent on these structural changes.

## 2. Results and Discussion

### 2.1. Synthesis

**Scheme 1:**
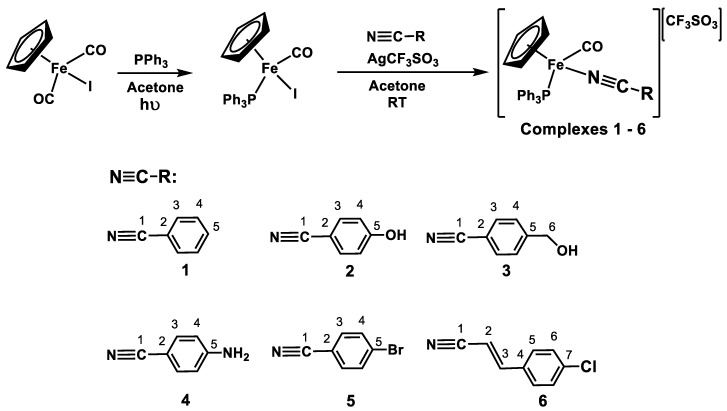
Reaction scheme for the synthesis of the starting material. [Fe(η^5^-C_5_H_5_)(CO)(PPh_3_)I] and for compounds **1**–**6**. Numbering of the ligands is for NMR purposes.

The new cationic complexes of the general formula [Fe(η^5^-C_5_H_5_)(CO)(PPh_3_)(NCR)]^+^ were obtained by iodide abstraction while using AgCF_3_SO_3_ followed by reaction with nitriles (NCR = benzonitrile (**1**); 4-hydroxybenzonitrile (**2**); 4-hydroxymethylbenzonitrile (**3**); 4-aminobenzonitrile (**4**); 4-bromobenzonitrile (**5**); 4-chlorocinnamonitrile (**6**)) and, in acetone at room temperature for 24 h (Scheme 1). Pure compounds were obtained in 65–79% yields, by column chromatography in neutral alumina, while using a mixture of acetone/*n*-hexane as eluent system for all complexes (*cf* experimental section). 

### 2.2. NMR Spectroscopy

NMR characterization of the aforementioned complexes was done by ^1^H, ^13^C{^1^H}-apt, ^31^P{^1^H}, HMQC, and HMBC. Table 1 presents the ^1^H NMR values of the Cp ligand and of the *ortho* protons of each benzonitrile (H3). As it can be observed, there is a deshielding on the Cp upon the coordination of the nitriles up to 0.64 ppm, as expected for ‘FeCp’ cationic complexes. Additionally, since benzonitriles are good π acceptors, it is possible to verify a common shielding effect on these ligand protons for all the complexes up to −0.45 ppm at H3, indicative of π-backdonation. Complex **3** has the most deshielded H3 proton, as it has an alkyl substituent at the *para* position of the phenyl group. This substituent is a ring activator, possibly leading to an elongation of the aromatic π-system. On the contrary, complex **5** has the most shielded H3 proton due to the presence of the electronegative bromine that pulls the electronic density. The 4-chlorocinnamonitrile ligand that was used for the synthesis of complex **6** was a mixture of *cis* (40%) and *trans* (60%) forms. The resulting complex maintained the same proportion between the two isomers, being easily identified by H2 and H3 coupling constants (*J_HH_* = 16 Hz for the *trans* isomer and *J_HH_* = 12 Hz for the *cis* isomer). In this case, the shieling effect is mostly felt at *trans*-H3 (−0.54 ppm). 

^13^C{^1^H} NMR data show the Cp ring at the usual chemical shift for monocationic Fe(II) compounds and a significant deshielding (up to +44 ppm) on the C1 (C≡N) upon nitriles coordination. The ^31^P{^1^H} spectrum is characterized by the presence of a single signal at 66 ppm for all of the compounds. 

### 2.3. FTIR Spectroscopy

The solid state FTIR spectra (KBr pellets) of all the complexes present the characteristic bands for the cyclopentadienyl ring along with the aromatic rings of the triphenylphosphane and nitrile ligands (3200–3000 cm^−1^ and 1600–1400 cm^−1^). Additional bands at ~1250, 1160, and 1029 cm^−1^ confirm the presence of the counter-ion (CF_3_SO_3_)^−^. The presence of coordinated carbonyl can be confirmed by the infrared stretching vibration, appearing at 1956–1996 cm^−1^. The replacement of the iodide in [Fe(η^5^-C_5_H_5_)(CO)(PPh_3_)I] by the nitrile ligand in compounds **1**–**6** leads to a strengthening of the (C≡O) bond (1935 vs. 1956–1996 cm^−1^) and it accounts for the π-backdonation competition with the other π-acceptor nitrile ligands (Table 2). It is also possible to observe a significant variation between complexes **5** and **6** with respect to the vibration frequency of the coordinated carbonyl, which was related with the nitrile substituent. Complex **5** is a benzonitrile substituted at the *para* position with a bromine, which is an electronegative atom that acts as an inducting withdrawing group. 

The coordination of the nitriles to the iron center can be confirmed by the characteristic stretching vibration of the nitrile functional group appearing at 2237–2250 cm^−1^ (Table 2). This variation of 16–25 cm^−1^ for the υ(N≡C) when compared with the free ligand accounts for the σ coordination and the effect of metal-ligand π-backdonation through d_metal−_π*_ligand_ interaction that was observed in other related compounds [23]. 

### 2.4. UV-VIS Spectroscopy

**Figure 2:**
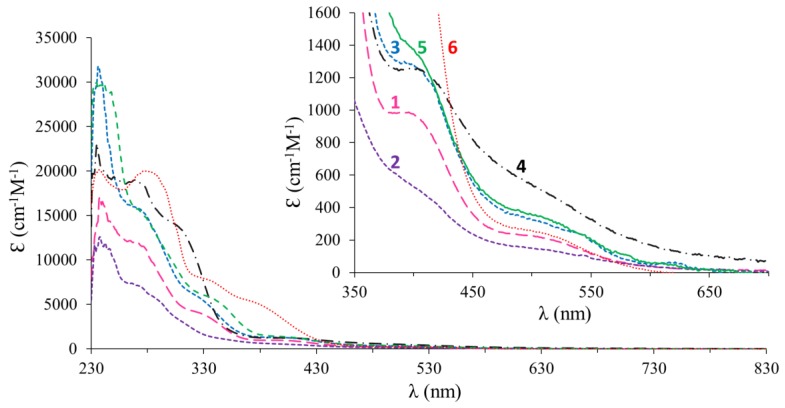
Electronic spectra of complexes **1**–**6** in DCM solution.

The UV-Visible study of the compounds **1**–**6** was performed in DCM and DMSO solutions (10^−3^–10^−5^ M) in the spectral window that was allowed by the solvents. All of the compounds have in common π→π* transitions below 285 nm, attributed to intraligand transitions (Figure 2), which usually occur in aromatic and chromophoric ligands, such as the phosphane and nitriles. One can also observe a shoulder at around 300–330 nm that was attributed to electronic transitions occurring in the organometallic fragment {FeCp}^+^ (by analogy with the iron parental complex [Fe(Cp)(CO)_2_I]) [20]. In addition, two bands/shoulders were found for all of the complexes. The first one around 400 nm, with values of ε ~1000 M^−1^cm^−1^, is probably a metal-to-ligand charge transfer band (MLCT), since the other techniques used (^1^H NMR and FT-IR) showed the presence of π-backdonation from the metal center to the nitrile and to the carbonyl; however, other contributions cannot be excluded. The second shoulder, above 500 nm, may be a d-d band, as it is common to all complexes and it has values of ε < 500 M^−1^cm^−1^. This point will be further clarified below (TD-DFT calculations).

### 2.5. Single-Crystal Structures of Compounds **1**, **4** and **5**

**Figure 3:**
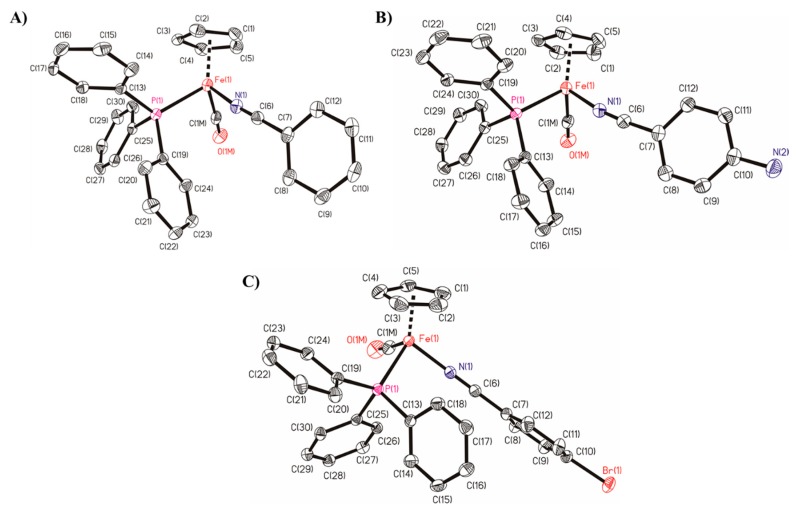
ORTEP for the cations of complexes (**A**) [Fe(η^5^-Cp)(CO)(PPh_3_)(NCPh)]^+^ **1**, (**B**) [Fe(η^5^-Cp)(CO)(PPh_3_)(p-NCPhNH_2_)]^+^ **4** and (**C**) [Fe(η^5^-Cp)(CO)(PPh_3_)(p-NCPhBr)]^+^ **5**. All of the non-hydrogen atoms are presented by their 50% probability ellipsoids. Hydrogen atoms are omitted for clarity.

Compounds [Fe(*η*^5^-Cp)(CO)(PPh_3_)(NCPh)][CF_3_SO_3_] **1**, [Fe(*η*^5^-Cp)(CO)(PPh_3_)(*p*-NCPhNH_2_)] [CF_3_SO_3_] **4**, and [Fe(*η*^5^-Cp)(CO)(PPh_3_)(*p*-NCPhBr)][CF_3_SO_3_] **5** crystallize from acetone/n-hexane solutions as orange blocks (crystal dimensions 0.23 × 0.14 × 0.07 mm), orange needles (crystal dimensions 0.15 × 0.10 × 0.04 mm), or orange plates (crystal dimensions 0.25 × 0.04 × 0.03 mm), respectively. Figure 3 shows the ORTEP representations of these compounds.

All the compounds crystallize in a centrosymmetric monoclinic, P2_1_c, space group. In the molecular structures, the iron centres adopt a “piano stool” distribution formed by the iron-Cp unit bound to one phosphine, one CO molecule, and one of different benzonitrile molecules. The C-O bond lengths are between 1.13–1.16 Å (see Table S1), and they decrease in the sequence *p*-H > *p*-NH_2_ > *p*-Br, which indicates that an increased contribution of resonance structure due to an increase in the character π-acceptor of the ligand and/or the effect of the electronegativity of *p*-substituent in benzonitrile molecules causes that the Fe back-bonding into the CO π* orbital is less important and the distance increases [24]. An increase of strong π-acceptor character in benzonitrile molecules, compete with CO molecule for the electron back-donation and the CO stretch frequencies remain high, as also observed by the FTIR stretching frequencies as well [25]. The distances between Fe and the centroids of the π-bonded cyclopentadienyl moiety to Fe centre are: 1.7158(17) Å (ring slippage 0.043 Å) in complex **1**, 1.7166(10) Å (ring slippage 0.046 Å) in complex **4,** and 1.7135(18) Å (ring slippage 0.042 Å) in complex **5**. The mean values of the Fe-C bond distances are: 2.095(4) in **1**, 2.096(2) Å in **4** and 2.099(4) Å in **5**. Table S1 contains selected bond lengths and angles for all of the compounds. 

**Figure 4:**
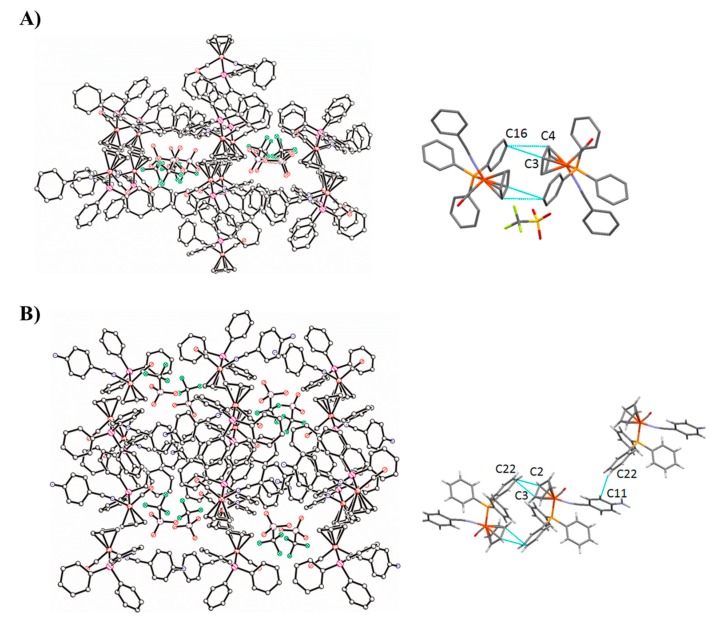
Crystal packing of the compounds (**A**) [Fe(η^5^-Cp)(CO)(PPh_3_)(NCPh)][CF_3_SO_3_)] **1** and (**B**) [Fe(*η*^5^-Cp)(CO)(PPh_3_)(*p*-NCPhNH_2_)][CF_3_SO_3_] **4** (left; drawing were done with SHELXTL program in balls and sticks). Short contacts between complexes are showed in dashed blue lines (right) (drawings were done with mercury 3.7 program in capped sticks). Hydrogen atoms are omitted for clarity.

**Figure 5:**
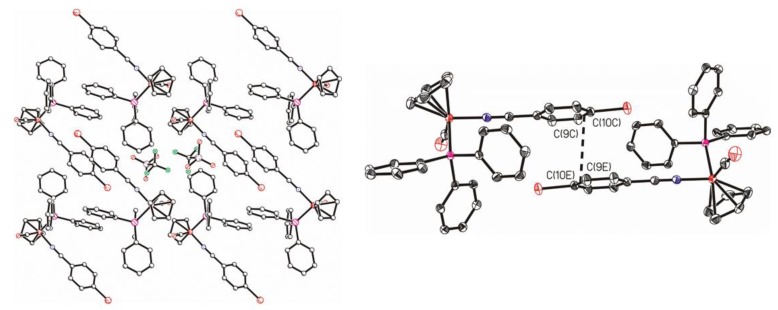
Crystal packing of the compound [Fe(η^5^-Cp)(CO)(PPh_3_)(p-NCPhBr)][CF_3_SO_3_] **5** (left; drawing were done with SHELXTL program by balls and sticks). Short contacts between complexes are showed in dashed black lines (right; drawing was done with SHELXTL program by their 50% probability ellipsoids). Hydrogen atoms are omitted for clarity.

Short contacts are observed between the phenyl rings of phosphane ligands and Cp rings (see Figure 4A). The distance C(3)-C(16) is 3.172(3) Å and C(4)-C(16) is 3.298(3) Å (through symmetry operation, 1 − x, −y, 2 − z) in complex **1** and C(2)-C(22) is 3.343(2) Å and C(3)-C(22) is 3.201(2) Å (through symmetry operation, -x,-y,-z) in complex **4**. CH-π-carbon atom of phosphine phenyl ring interaction appears in the crystal packing of complex **4** [distance C(11)-H(22): 2.842(2) Å] (see Figure 4). A π-π lateral interaction is observed between NCPh-*ρ*-Br rings in complex **5** (see Figure 5), which is the predominant interaction and determines the crystal packing in this compound. The distance between centroids, d_c1-c2_, is 3.531(2) Ǻ [c1, C(9C)-C(10C), c2, C(9E)-C(10E)]. Hydrogen bonds are present in the structure of *p*NH_2_-derivative (**4**). Intermolecular hydrogen bond between N(2)-O(3) [d(D-H): 0.96(3) Å, d(D...A): 2.21(3) Å, d(D...A): 3.112(3)Å and <(DHA): 157(2)°, symmetry transformation: −x, y − 1/2, −z + 3/2] is the unique that is observed. Different types of intermolecular interactions can also occur when these compounds interact with biomolecules and they can justify their different biological activities.

### 2.6. TD-DFT Calculations

**Figure 6:**
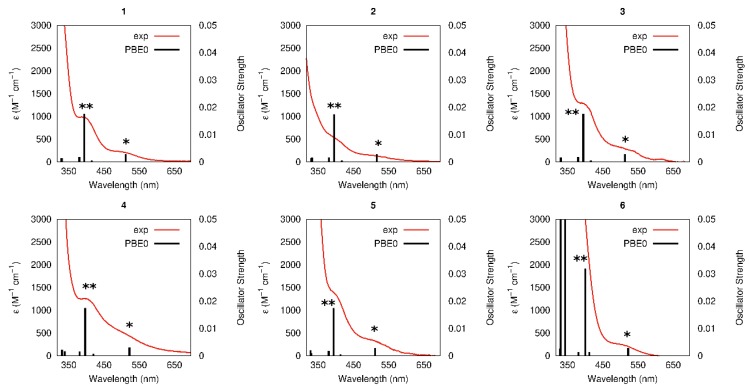
TD-DFT excitations (black lines) plotted against the UV−visible absorption spectra (red) in dichloromethane for complexes **1**–**6**. The excitations reported in Table S2 are highlighted (*/**).

TD-DFT calculations (PBE0) were performed in dichloromethane for all complexes to help in the interpretation of the UV-Vis. data (see computational details). Table S2 lists the relevant calculated excitations. The TD-DFT excitations reproduce the two low-energy shoulders that were experimentally observed for all complexes (>500 nm and ~400 nm) with a perfect quantitative agreement. Indeed, the superposition of the experimental spectra with the calculated excitations, as shown in Figure 6, is remarkable.

**Figure 7:**
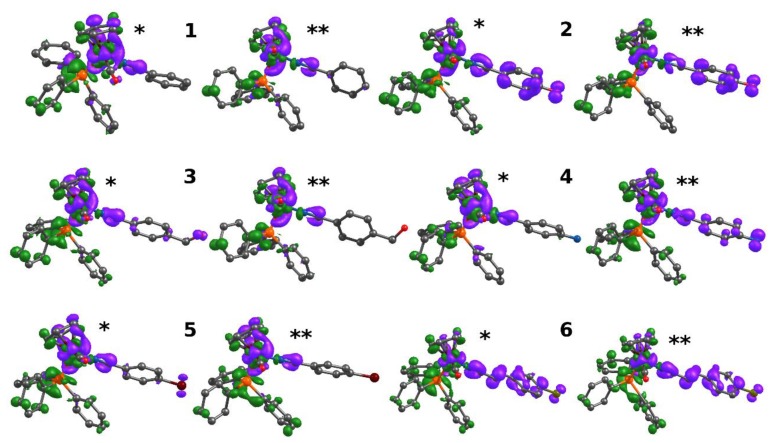
Electron density difference maps (EDDM) for complexes **1**–**6** for excitations presented in Table S2 and highlighted in Figure 6 with * and **. Violet and green correspond to a decrease and increase of electron density, respectively.

In all complexes, the low-energy excitations are dominated by two transitions (* and ** in Figure 6) whose compositions are highly mixed (Table S2), with contributions from several orbitals. Electron density difference maps (EDDM), which represent the changes in electron density upon excitation, were also obtained in order to assist the assignment of the transitions (Figure 7). Surprisingly, for most complexes, the two low-energy excitations have very similar character apart from complex **4**, where slightly different EDDMs are observed (see below). For complexes **1**, **3**, and **5**, both of the excitations have d-d character mixed with charge transfer from iron center (and NC bond) to the PPh_3_ ligand (MLCT). On the other hand, for complexes **2** and **6**, besides the mixed d-d/MLCT character, there is a clear participation of the nitrile ligand, i.e., there is a charge transfer from Fe-nitrile ligand to the PPh_3_ ligand. In complex **4**, the lowest-energy excitation resembles the ones that were obtained for **1**, **3**, and **5**, whereas the second excitation is similar to the ones observed for **2** and **6** with the participation of the nitrile ligand.

### 2.7. Biological Assays

#### 2.7.1. Stability Assays

For the intended application as anticancer agents, it is desirable that the compounds under study are stable in cellular medium. Thus, the stability of these compounds has been tested by UV-Vis. spectroscopy in 100% DMSO (Figure S37), which is the co-solvent that is used for the biological studies, and in culture cellular media (DMEM) while using 5% of DMSO (Figure S38). Under these conditions, all of the compounds were very stable up to 24 h, with absorbance variations of less than 5%.

#### 2.7.2. In Vitro Cytotoxicity Evaluation and IC_50_ Determination

The cytotoxic activity of these new [Fe(η^5^-C_5_H_5_)(CO)(PPh_3_)(NCR)][CF_3_SO_3_] compounds (abrev. ‘FeCp’) was assessed in the triple negative breast cancer-derived cell line MDA-MB-231, in the colorectal cancer-derived cell line SW480 and in the noncancerous cell line NCM460, derived from normal colon epithelial cells. All of the cells were incubated for 48 h with different concentrations of all iron compounds. All the nitrile organic ligands were also tested in the MDA-MB-231 cell line. These cell lines were chosen due to the high incidence and prevalence of these cancers and since there is no currently available treatments for them. Indeed, breast and colorectal cancers are among the top three causes of cancer death worldwide. All of the complexes are very active compounds in the colorectal cancer cells SW480, as well as for breast cancer cells MDA-MB-231 (Table 3). None of the nitrile ligands was cytotoxic up to 100 µM. All of the ‘FeCp’ compounds affected the cell growth of SW480 and MDA-MB-231 cell lines in the micromolar range, being more cytotoxic for the breast cancer cells. The noncancerous cell line NCM460 was shown to be more resistant to the treatment with the ‘FeCp’ compounds. The half-maximal inhibitory concentration (IC_50_) that was determined for SW480 cell line, for all compounds, was half of the IC_50_ values determined in the “normal” cell line NCM460. These results suggest that these compounds are more cytotoxic in cancer cells than in “normal” cells (Table 3), showing a higher IC_50_ for NCM460 cell line, for all six compounds. These results are a good starting point, since it is expected that, during a chemotherapy cycle, the dosage of the drug should kill cancer cells without affecting the normal and healthy cells of the organism. The selectivity index (SI) is an index value that gives an idea about the selectivity of a compound. For almost all of the compounds, the selectivity indices were equal or greater than two (Table 3). Some authors consider this value to be an interesting selectivity index, meaning that the compound show the double of the cytotoxic to the tumor cells when comparing to the normal cells [26]. The obtained results are in line with what is intended in the clinics, since they suggest that the dose used to affect tumor cells will not affect the normal and healthy cells of the patient. 

When comparing these results with related ‘RuCp’ compounds, similar IC_50_ values were observed for the SW480 cell line [17], while, for the MDA-MB-231 cell line, the new compounds here reported seem to show better cytotoxicity [14]. Although direct comparison with other ‘FeCp’ compounds is not possible due to differences in cell lines and incubation times, the results from the literature for [Fe(Cp)(dppe)(nitrile carbohydrate derivatives)]^+^ in the colon cancer HCT116 cell line (72 h incubation) [27] indicate that the results reported here are very promising. Additionally, regarding our previous work on similar compounds with different substituents on the phosphane ligands, [Fe(η^5^-Cp)(CO)(phosphane)(aminobenzonitrile)]^+^ [20], one can infer that the changes introduced at the nitrile ligand seem to impart better cytotoxicity.

#### 2.7.3. Iron Compounds Reduce the Colony Formation Ability of SW480 Colorectal Cancer Cells

**Figure 8:**
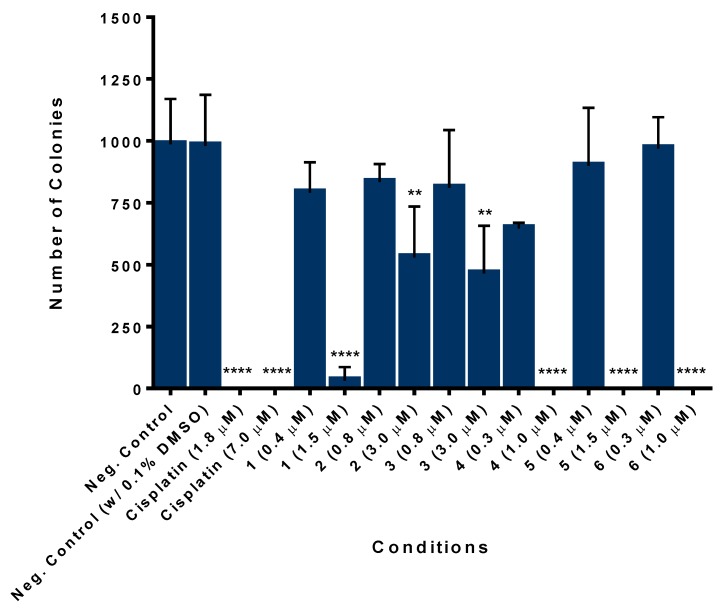
Iron compounds affect the colony formation ability of SW480 cell line. Analysis of the colony formation ability, after 48 h of incubation with ¼ IC_50_ and IC_50_, in SW480 cell line. The values represent mean ± S.D. of at least three independent experiments. Statistical analysis was performed by one-way ANOVA with Dunnett’s multiple comparisons test. ** *P* ≤ 0.01; **** *P* ≤ 0.0001 compared with negative control.

The lack of success of some chemotherapeutic treatments is due to the fact that, even after several cycles of chemotherapy, some cells may relapse and maintain their malignant potential. In this way, the effects on cell survival and proliferation of iron-based compounds was assessed by colony formation assay. This technique allows for determining the cellular ability to survive to the exposure of an exogenous agent for a short period of time and produce colonies after that agent is removed, simulating in vitro what happens during cycles of chemotherapy. The SW480 colorectal cancer cell line was incubated with ¼ IC_50_ and IC_50_ concentrations of the Fe compounds, during 48 h. Cisplatin was used as the positive control. The results showed that the conditions that were treated with the IC_50_ values of Fe compounds have significantly affected the colony formation ability of SW480 cells (Figure 8 and Figure S39). Regarding cisplatin, in both conditions, the compound completely inhibited the formation of colonies. The compounds with the lowest IC_50_ values (Table 3) are those that are inhibiting the ability to form colonies to a large extent (**1**, **4**, **5**, and **6**; assays done at IC_50_).

#### 2.7.4. [Fe(η^5^-C_5_H_5_)(CO)(PPh_3_)(NCR)][CF_3_SO_3_] Compounds Induce Apoptosis in SW480 Colorectal Cancer-Derived Cell Line

**Figure 9:**
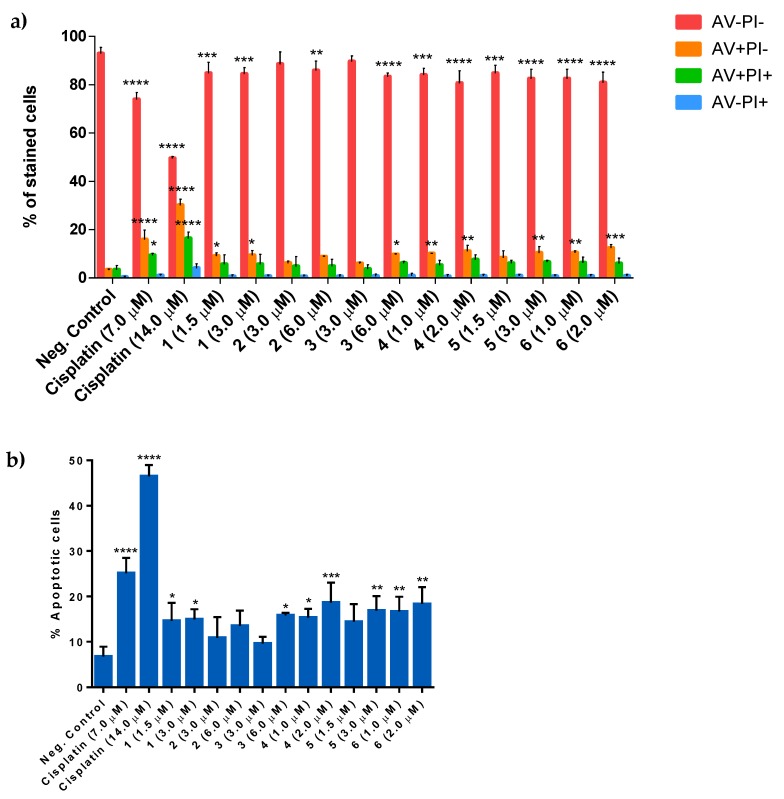
‘FeCp’ compounds induce apoptosis in SW480 colorectal cancer-derived cell line. Apoptotic cell death was analyzed by Annexin V fluorescein isothiocyanate (AV-FITC) and propidium iodide (PI) assay in SW480 cells, after incubation with IC_50_ and 2 × IC_50_ concentrations for 48 h. (**a**) Analysis of AV/PI assay in SW480 cells. Values represent mean ± S.D. of at least three independent experiments. Statistical analysis was performed by two-way ANOVA with Dunnett’s multiple comparisons test. (**b**) Graphical representation of the total percentage of apoptotic cells (early apoptosis (AV + PI^−^) and late apoptosis (AV + PI^+^)). Statistical analysis was performed by one-way ANOVA with Dunnett’s multiple comparisons test. * *P* ≤ 0.05; ** *P* ≤ 0.01; *** *P* ≤ 0.001; **** *P* ≤ 0.0001 when compared with the negative control.

The cell death mechanism of iron compounds was determined using Annexin V/Propidium iodide (AV/PI) cytometry-based assay. Annexin V binds with high affinity to the phospholipid phosphatidylserine (PS) that was present in the membrane. PS translocation from the inner side of the plasma membrane to the surface is an early event of apoptosis (AV + PI^−^). On the other hand, when the integrity of the membrane is compromised, PI binds with DNA, allowing for the distinction between late apoptosis (AV + PI^+^) or necrosis (AV−PI^+^). The SW480 cells were incubated with the compounds, for 48 h, at their IC_50_ and 2 × IC_50_ values and cisplatin was used as the positive control. The results showed that incubation with the ‘FeCp’ compounds led to an increase in the percentage apoptotic cells (AV + PI^−^ and AV + PI^+^) (Figure 9a and Figure S40). This is more evident when we put together the percentage of early apoptotic cells (AV + PI^−^) and the late apoptotic cells (AV + PI^+^), which correspond to the total levels of apoptosis (see Figure 9b). We could observe that, with the exception of compound **2**, all of the ‘FeCp’ compounds induce apoptosis at statistically significant levels when comparing with the negative control, although compounds **3** and **5** only reach statistical significance with the 2 × IC_50_ dose. We can also observe a dose/effect for compounds **3**, **4**, and **5**, although not so evident as cisplatin. We also showed that the conditions that were treated with cisplatin had higher levels of apoptosis than conditions incubated with ‘FeCp’ compounds. Regarding necrotic cells, these cells are represented by AV−PI^+^ staining (in blue), and, as observed in Figure 9a, there is a low percentage of necrotic cells induced by the compounds which are not significantly different from the negative control. Therefore, these results indicate that ‘FeCp’ compounds do not induce necrosis. Overall, compounds **1**, **4**, **5**, and **6**, in addition to inhibiting the formation of colonies and having the lowest IC_50_ values, they also induce apoptosis in a significant manner. Thus, these compounds stand out from the remaining compounds due to their promising results. 

#### 2.7.5. [Fe(η^5^-C_5_H_5_)(CO)(PPh_3_)(NCR)][CF_3_SO_3_] Compounds Affect the Actin Cytoskeleton of SW480 Colorectal Cancer Cells

**Figure 10:**
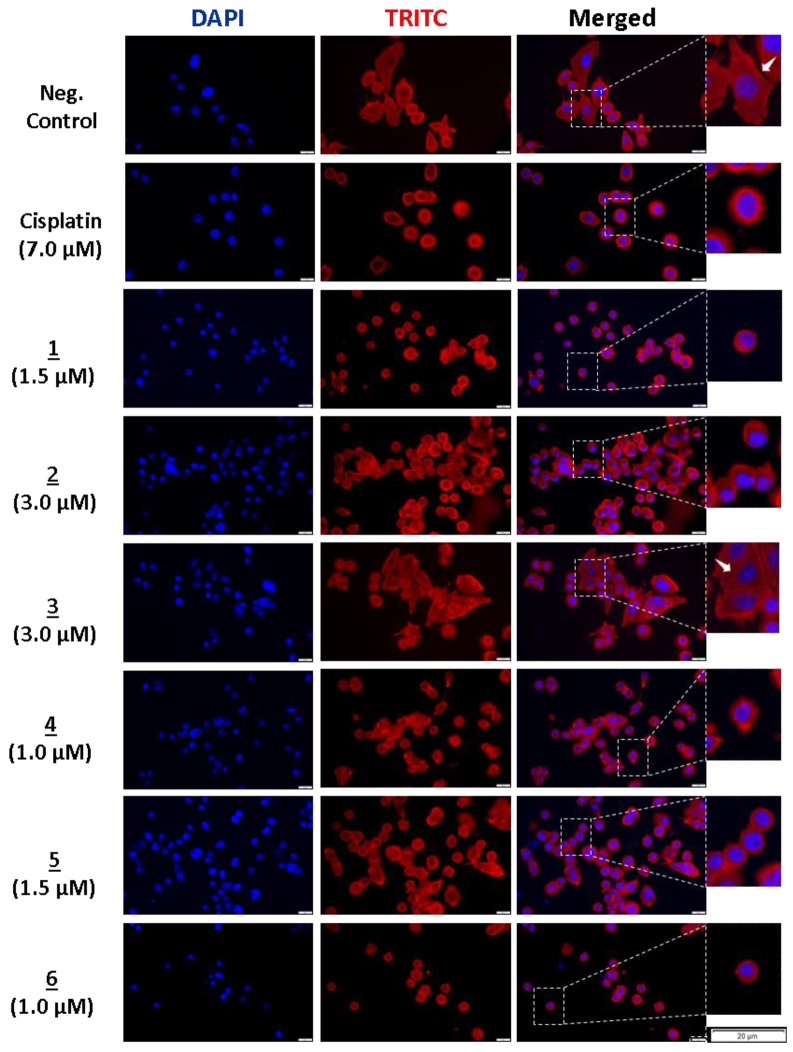
‘FeCp’ compounds affect the cytoskeleton of SW480 colorectal cancer-derived cell line. The analysis of F-actin staining was performed using the IC_50_ values of each compound, for 48 h. Representative images (×600) of DAPI (4’,6-diamidino-2-phenylindole), Phalloidin-AlexaFluor^®^ 568 and merged were obtained in a fluorescence microscope. The results were obtained from at least three independent experiments.

With the purpose of evaluating the effects of these new compounds in the cytoskeleton structure of SW480 colorectal cancer-derived cell line, the F-actin organization and morphology was analyzed while using phalloidin. The cells were treated with IC_50_ concentrations of each compound, for 48 h, and then were stained with Phalloidin-AlexaFluor^®^ 568. In the negative control, we observed that cells are bigger, with a well-marked and enlarged cytoskeleton around the nucleus. We can also distinguish the establishment of connections between cells showing filipodia-like structures. In the conditions that were treated with cisplatin and compounds **1**, **2**, **4**, **5**, and **6**, the cells are smaller and rounded, losing the filipodia-like structures (Figure 10). In these conditions, we observed that the cytoskeleton is just around the nucleus in a thin line. Cisplatin and compounds **1**, **4**, and **6** also decreased the cell number, showing an increase in the distance between cells affecting the cell-cell adhesions and intercellular contacts establishment, thus decreasing the communication between cells. Compound **3** affected the actin cytoskeleton in a less perceptible way, decreasing the size of some cells and leading to a rounding phenotype of others. In general, the results showed that the compounds with the lowest IC_50_ values have a pronounced effect on the inhibition of colony formation, an increase in apoptosis with low doses of the compounds, and alteration on the actin cytoskeleton morphology suggesting a cytotoxic effect of these agents in SW480 colorectal cells.

## 3. Materials and Methods

All of the reactions and organometallic synthesis were carried out under nitrogen atmosphere while using standard Schlenk techniques. The solvents used were previously and they were freshly distilled under nitrogen atmosphere before use, according to common literature methods. The NMR spectra were recorded on a Brucker Advance 400 spectrometer (Fällanden, Switzerland) (^1^H, 400 MHz; ^13^C{^1^H}-apt, 100.62 MHz; ^31^P{^1^H}, 161.97 MHz) at probe temperature. ^1^H and ^13^C chemical shifts were reported downfield from the residual solvent peak, whereas the ^31^P NMR chemical shifts were reported downfield from the external standard 85% H_3_PO_4_. All of the resonances were characterized for their chemical shifts (*δ*), given in parts-per-million (ppm), and for their coupling constants (*J*) expressed in Hertz (Hz). Resonance multiplicity is expressed, as follows: singlet (s), doublet (d), triplet (t), multiplet (m), and complex (comp). All of the assignments were attributed using HMBC, HMQC, and COSY 2D-RMN techniques. Each sample was prepared under air and at room temperature, while using the most adequate deuterated solvent. The electronic UV-Vis spectra were recorded in dichloromethane and dimethylsulfoxide solutions (10^−3^–10^−5^ M), under air, using 1 cm optical path quartz cells on a Jasco V-660 spectrometer (Elnor, Porto, Portugal) in the range of 200–900 nm. The infrared spectra were recorded in a Shimadzu IRAffinity-1 FTIR spectrophotometer (Kyoto, Japan) in dry KBr pellets, under air, and at room temperature. Elemental analyses were obtained at *Laboratório de Análises, Instituto Superior Técnico*, using a Fisons Instruments EA1108 system (Fison Instruments Ltd., Glasgow, UK). Data acquisition, integration, and handling were performed using a PC with the software package EAGER-200 (Carlo Erba Instruments).

For the thin layer chromatography (TLC-PET) analytical assays, aluminum oxide on PET of several appropriate dimensions was used. The elution of these plates was performed while using different proportions of acetone/n-hexane. Upon elution, the TLC plates were observed under UV light at 254 and 366 nm.

### 3.1. Synthesis

The starting material, [Fe(η^5^-C_5_H_5_)(CO)_2_I], was prepared from the commercially available dimer following the literature procedure [28].

#### 3.1.1. Synthesis of [Fe(η^5^-C_5_H_5_)(CO)(PPh_3_)(I)]

The synthesis of the complex [Fe(η^5^-C_5_H_5_)(CO)(PPh_3_)I] has been previously reported [20,24]. Yet, in this protocol, we increased the yield from 69 to 75% [20]

To a stirred and degassed solution of [Fe(η^5^-C_5_H_5_)(CO)_2_I] (1.8 mmol) in dry acetone (40 mL), PPh_3_ (1.8 mmol) was added. The reaction mixture was then irradiated under UV light of 300 W for 3 h. The solvent was removed under vacuum and the obtained product was dissolved in the minimum quantity of dry acetone to remove the soluble impurities. A green powder precipitated (the product) and the impurity was separated by cannula-filtration. The residue was twice recrystallized from dry acetone/n-hexane and dark green products are obtained. 

Yield: 80%. Green powder. The obtained NMR results are in agreement with the values that were found in the literature [20] FTIR (KBr, cm^−1^): υ(C-H aromatics) 3100–3000, υ(C≡O) 1935, υ(C-C aromatics) 1600–1400. UV-Vis in DMSO, λ_max_/nm [ε/M^−1^ cm^−1^]: 275 (*Sh*); 325 (2470); 387 (*Sh*); 448 (760); 626 (155). UV-Vis in DCM, λ_max_/nm [ε/M^−1^ cm^−1^]: 237 (21000); 241 (22765); 326 (2686); 445 (800); and, 618 (200). 

#### 3.1.2. General Procedure for the Synthesis of [Fe(η^5^-C_5_H_5_)(CO)(PPh_3_)(NCR)][CF_3_SO_3_] Complexes **1**–**6**

AgCF_3_SO_3_ (0.8 mmol) and the respective nitrile (0.5 mmol) were added to a stirred and degassed solution of [Fe(η^5^-C_5_H_5_)(CO)(PPh_3_)I] (0.5 mmol) in dry acetone (30 mL) and the reaction was followed for 24 h at room temperature, monitored by ^1^H and ^31^P NMR or TLC. The precipitates were separated by cannula-filtration and the solvent was evaporated under vacuum. The product is purified by column chromatography, always using a mixture of acetone/*n*-hexane with different proportions, according to each product.

#### 3.1.3. [Fe(η^5^-C_5_H_5_)(CO)(PPh_3_)(NCPh)][CF_3_SO_3_]—Complex **1**

Yield: 69%, Red crystalline powder. Eluent: acetone/*n*-hexane (1:1).

^1^H NMR [(CD_3_)_2_CO-d_6_, Me_4_Si, δ/ppm]: 7.70 (t, 1, H5); 7.66–7.57 (comp, 9, *H_meta_*+*H_para_*-PPh_3_); 7.53–7.49 (m, 6, *H_ortho_*-PPh_3_); 7.37 (d, 2, H3); 7.3 (t, 2, H4); 5.21 (s, 5, (η^5^-C_5_H_5_)). ^13^C NMR [(CD_3_)_2_CO-d_6_, Me_4_Si, δ/ppm]: 216.35 (d, *J*_CP_ = 28, C≡O); 157.24 (C1); 135.38 (C2); 134.09 (C5); 133.02 (d, ^2^*J*_CP_ = 10, *C_ortho_*-PPh_3_); 132.45 (C3); 131.92 (d, ^1^*J*_CP_ = 46, *C_ipso_*-PPh_3_); 131.22 (d, ^4^*J*_CP_ = 3, *C_para_*-PPh_3_); 129.09 (d,^3^*J*_CP_ = 10, *C_meta_-*PPh_3_); 128.97 (C4); 85.15 (η^5^-C_5_H_5_). ^31^P NMR [(CD_3_)_2_CO-d_6_, H_3_PO_4_, δ/ppm]: 66.55. FTIR [KBr cm^−1^]: υ(C-H aromatics) 3150, 3091, 3050; υ(N≡C) 2247; υ(C≡O) 1977; υ(CF_3_SO_3_^−^) 1257, 1157, 1029. UV-Vis in DMSO, λ_max_/nm[ε/M^−1^cm^−1^]: 272 (11092); 275 (11093); 278 (11093); 282 (*Sh*); 285 (*Sh*); 290 (*Sh*); 331 (*Sh*); 401 (*Sh*); 501 (*Sh*). UV-Vis in DCM, λ_max_/nm[ε/M^−1^cm^−1^]: 235 (14060); 238 (17100); 240 (16560); 247 (14300); 269 (12130); 286 (9500); 335 (4220); 390 (990); 502 (225). ESI-MS (+, *m*/*z*) Calc for [**1**]^+^: 514.10. Found: 513.66. Elemental analysis (%) Calc. for C_32_H_25_F_3_FeNO_4_PS. C 57.9; H 3.7; N 2.1; S 4.8; Found: C 57.6; H 3.6; N 1.8; S 5.0.

#### 3.1.4. [Fe(η^5^-C_5_H_5_)(CO)(PPh_3_)(p-NCPhOH)][CF_3_SO_3_]—Complex **2**

Yield: 66%. Red crystalline powder. Eluent: acetone/*n*-hexane (3:1). An increasing proportion of acetone is used until the product is completely extracted.

^1^H NMR [(CD_3_)_2_CO-d_6_, Me_4_Si, δ/ppm]: 9.82 (s, 1, OH); 7.66–7.56 (comp, 9, *H_meta_*+*H_para_*-PPh_3_); 7.53 (t, 6, *H_ortho_*-PPh_3_); 7.19 ( d, *J*_HH_ = 20, H3); 6.90 (d, *J*_HH_ = 20, H4); 5.17 (s, 5, (η^5^-C_5_H_5_)). ^13^C NMR [(CD_3_)_2_CO-d_6_, Me_4_Si, δ/ppm]: 217.66 (d, *J*_CP_ = 28, C≡O); 163.64 (C1); 137.45 (C2); 135.48 (C3); 134.06 (d, ^2^*J*_CP_ = 10, *C_ortho_*-PPh_3_); 133.11 (d, ^1^*J*_CP_ = 46, *C_ipso_*-PPh_3_); 132.25 (d, ^4^*J*_CP_ = 3, *C_para_*-PPh_3_); 130.12 (d, ^3^*J*_CP_ = 10, *C_meta_-*PPh_3_); 117.16 (C4); 101.53 (C5); 86.01 (η^5^-C_5_H_5_). ^31^P NMR [(CD_3_)_2_CO-d_6_, H_3_PO_4_, δ/ppm]: 66.62. FTIR [KBr cm^−1^]: υ(O-H) 3217; υ(C-H aromatics) 3080, 2962; υ(N≡C) 2249; υ(C≡O) 1986; υ(CF_3_SO_3_^−^) 1261, 1160, 1026. UV-Vis in DMSO, λ_max_/nm[ε/M^−1^cm^−1^]: 273 (16461); 278 (16075); 288 (*Sh*); 330 (*Sh*); 408 (*Sh*); 507 (*Sh*). UV-Vis in DCM, λ_max_/nm[ε/M^−1^cm^−1^]: 238 (12575); 242 (11880); 247 (11150); 250 (10100); 267 (7320); 274 (7010); 293 (5080); 313 (2860); 407 (492); 507 (143); 546 (103). ESI-MS(+, *m*/*z*) Calc for [**2**]^+^: 530.09. Found: 529.68. Elemental analysis (%) Calc. for C_32_H_25_F_3_FeNO_5_PS. C 56.5; H 3.7; N 2.0; S 4.7; Found: C 54.5; H 3.6; N 2.0; S 5.0;

#### 3.1.5. [Fe(η^5^-C_5_H_5_)(CO)(PPh_3_)(p-NCPhCH_2_OH)][CF_3_SO_3_]—Complex **3**

Yield: 65%. Red crystalline powder. Eluent: acetone/*n*-hexane (2:1). An increasing proportion of acetone is used until the product is completely extracted.

^1^H NMR [(CD_3_)_2_CO-d_6_, Me_4_Si, δ/ppm]: 8.61 (s, 1, OH); 7.65–7.58 (comp, 9, *H_meta_*+*H_para_*-PPh_3_); 7.54 (t, 6, *H_ortho_*-PPh_3_); 7.48 (d, *J*_HH_ = 20, H3); 7.30 (d, *J*_HH_ = 10, H4); 5.20 (s, 5,(η^5^-C_5_H_5_)); 4.85 (s, 1, OH). ^13^C NMR [(CD_3_)_2_CO-d_6_, Me_4_Si, δ/ppm]: 217.35 (d, *J*_CP_ = 28, C≡O); 153.04 (C1); 136.66 (C2); 133.95 (d, ^2^*J*_CP_ = 10, *C_ortho_*-PPh_3_); 133.25 (C3); 132.80 (d, ^1^*J*_CP_ = 46, *C_ipso_*-PPh_3_); 132.17 (d, ^4^*J*_CP_ = 2, *C_para_*-PPh_3_); 130.00 (d,^3^*J*_CP_ = 10, *C_meta_-*PPh_3_); 127.40 (C4); 109.40 (C5); 86.01(η^5^-C_5_H_5_); 63.81 (C6). ^31^P NMR [(CD_3_)_2_CO-d_6_, H_3_PO_4_, δ/ppm]: 66.51. FTIR [KBr cm^−1^]: υ(O-H) 3450; υ(C-H aromatics) 3109, 2954; υ(C-H aliphatic) 2870; υ(N≡C) 2250; υ(C≡O) 1984; υ(CF_3_SO_3_^−^) 1273, 1161, 1029. UV-Vis in DMSO, λ_max_/nm[ε/M^−1^cm^−1^]: 274 (6050); 278 (5995); 285 (*Sh*); 289 (*Sh*); 331 (*Sh*); 403 (563); 502 (*Sh*). UV-Vis in DCM, λ_max_/nm[ε/M^−1^cm^−1^]: 236 (31670); 240 (29770); 271 (15930); 325 (5865); 397 (1290); 546 (217); 623 (54). ESI-MS(+, *m*/*z*) Calc for [**3**]^+^: 544.11. Found: 543.67. Elemental analysis (%) Calc. for C_33_H_27_F_3_FeNO_5_PS. C 57.1; H 3.9; N 2.0; S 4.6; Found: C 56.4; H 3.7; N 1.8; S 5.0;

#### 3.1.6. [Fe(η^5^-C_5_H_5_)(CO)(PPh_3_)(p-NCPhNH_2_)][CF_3_SO_3_]—Complex **4**

Yield: 79%. Red crystalline powder. Eluent: acetone/*n*-hexane (2:1).

^1^H NMR [(CD_3_)_2_CO-d_6_, Me_4_Si, δ/ppm]: 7.64–7.57 (comp, 9, *H_meta_*+*H_para_*-PPh_3_); 7.55 (t, 6, *H_ortho_*-PPh_3_); 6.97 (d, *J*_HH_ = 20, H3); 6.64 (d, *J*_HH_ = 10, *H_ortho_*-PPh_3_); 5.23 (s, 2, NH_2_); 5.13 (s, 5,(η^5^-C_5_H_5_)). ^13^C NMR [(CD_3_)_2_CO-d_6_, Me_4_Si, δ/ppm]: 217.30 (d, *J*_CP_ = 28, C≡O); 154.14 (C1); 138.30 (C2); 134.46 (C3); 133.46 (d, ^2^*J*_CP_ = 10, *C_ortho_*-PPh_3_); 132.66 (d, ^1^*J*_CP_ = 47, *C_ipso_*-PPh_3_); 131.62 (d, ^4^*J*_CP_ = 2, *C_para_*-PPh_3_); 129.50 (d,^3^*J*_CP_ = 10, *C_meta_-*PPh_3_); 113.86 (C4); 95.69 (C5); 85.31 (η^5^-C_5_H_5_). ^31^P NMR [(CD_3_)_2_CO-d_6_, H_3_PO_4_, δ/ppm]: 66.68. FTIR [KBr cm^−1^]: υ(N-H) 3415; υ(C-H aromatics) 3238, 3108, 2985; υ(N≡C) 2237; υ(C≡O) 1980; υ(CF_3_SO_3_^−^) 1257, 1172, 1029. UV-Vis in DMSO, λ_max_/nm[ε/M^−1^cm^−1^]: 273 (*Sh*); 278 (18759); 283 (18924); 290 (17987); 320 (*Sh*); 404 (*Sh*); 520 (*Sh*). UV-Vis in DCM, λ_max_/nm[ε/M^−1^cm^−1^]: 235 (23100); 253 (19240); 271 (19000); 304 (14070); 406 (1245); 525 (440). ESI-MS (+, *m*/*z*) Calc for [**4**]^+^: 529.11. Found: 528.71. Elemental analysis (%) Calc. for C_32_H_26_F_3_FeN_2_O_4_PS. C 56.6; H 3.9; N 4.1; S 4.7; Found: C 53.4; H 4.0; N 3.5; S 5.0.

#### 3.1.7. [Fe(η^5^-C_5_H_5_)(CO)(PPh_3_)(p-NCPhBr)][CF_3_SO_3_]—Complex **5**

Yield: 72%, Red crystalline powder. Eluent: acetone/*n*-hexane (1:1).^1^H NMR [(CD_3_)_2_CO-d_6_, Me_4_Si, δ/ppm]: 7.72 (d, *J*_HH_ = 20, H4); 7.66–7.58 (comp, 9, *H_meta_*+*H_para_*-PPh_3_); 7.53 (t, 6, *H_ortho_*-PPh_3_); 7.31 (d, *J*_HH_ = 20, H3); 5.21 (s, 5, (η^5^-C_5_H_5_)). ^13^C NMR [(CD_3_)_2_CO-d_6_, Me_4_Si, δ/ppm]: 217.14 (d, *J*_CP_ = 27, C≡O); 135.45 (C1); 134.97 (C4); 133.95 (d, ^2^*J*_CP_ = 10, *C_ortho_*-PPh_3_); 133.23 (C3); 132.74 (d, ^1^*J*_CP_ = 46, *C_ipso_*-PPh_3_); 132.17 (d, ^4^*J*_CP_ = 2, *C_para_*-PPh_3_); 130.02 (d,^3^*J*_CP_ = 10, *C_meta_-*PPh_3_); 129.61 (C2); 110.56 (C5); 86.11 (η^5^-C_5_H_5_). ^31^P NMR [(CD_3_)_2_CO-d_6_, H_3_PO_4_, δ/ppm]: 66.51. FTIR [KBr cm^−1^]: υ(C-H aromatics) 3089, 3062; υ(N≡C) 2245; υ(C≡O) 1996; υ(CF_3_SO_3_^−^) 1270, 1157, 1029. UV-Vis in DMSO, λ_max_/nm[ε/M^−1^cm^−1^]: 272 (16775); 275 (16600); 278 (*Sh*); 281 (15797); 288 (*Sh*); 337 (*Sh*); 406 (*Sh*); 506 (354). UV-Vis in DCM, λ_max_/nm[ε/M^−1^cm^−1^]: 235 (29900); 240 (29685); 242 (29650); 248 (28870); 273 (15540); 279 (14735); 340 (5530); 404 (1337); 515 (323). ESI-MS(+, *m*/*z*) Calc for [**5**]^+^: 592.01. Found: 591.65. Elemental analysis (%) Calc. for C_31_H_24_BrF_3_FeNOP. C 51.7; H 1.8; N 1.9; S 4.3; Found: C 50.6; H 3.4; N 1.6; S 4.0;

#### 3.1.8. [Fe(η^5^-C_5_H_5_)(CO)(PPh_3_)(p-NCCH=CHPhCl)][CF_3_SO_3_]—Complex **6**

Yield: 75%, Red crystalline powder. Eluent: acetone/*n*-hexane (1:1).

FTIR [KBr cm^−1^]: υ(C-H aromatics) 3057, 2962; υ(N≡C) 2241; υ(C≡O) 1986; υ(CF_3_SO_3_^−^) 1253, 1170, 1029. UV-Vis in DMSO, λ_max_/nm[ε/M^−1^cm^−1^]: 274 (12115); 278 (12313); 282 (12095); 285 (*Sh*); 297 (*Sh*); 306 (*Sh*); 334 (*Sh*); 380 (*Sh*); 507 (*Sh*). UV-Vis in DCM, λ_max_/nm[ε/M^−1^cm^−1^]: 235 (23100); 244 (19645); 252 (19260); 258 (18840); 271 (19000); 278 (18215); 311 (13339); 409 (1233); 532 (408). ESI-MS(+, *m*/*z*) Calc for [**6**]^+^: 574.07. Found: 573.65. Elemental analysis (%) Calc. for C_34_H_26_ClF_3_FeNO_4_PS C 56.4; H 3.62; N 1.9; S 4.0; Found: C 57.2; H 3.4; N 1.7; S 4.0.

#### 3.1.9. [Fe(η^5^-C_5_H_5_)(CO)(PPh_3_)(trans-NCCH=CHPhCl)][CF_3_SO_3_]

^1^H NMR [(CD_3_)_2_CO-d_6_, Me_4_Si, δ/ppm]: 7.72–7.44 (comp, 19H, PPh_3_+H5+H6); 7.04 (d, *J*_HH_ = 16, H3); 6.26 (d, *J*_HH_ = 16, H2); 5.14 (s, 5, (η^5^-C_5_H_5_)). ^13^C NMR [(CD_3_)_2_CO-d_6_, Me_4_Si, δ/ppm]: 217.50 (d, *J*_CP_ = 26, C≡O); 152.92 (C3); 138.12 (C7); 136.47 (C4); 134.16 (d, ^2^*J*_CP_ = 10, *C_ortho_*-PPh_3_); 133.05 (d, ^3^*J*_CP_ = 26, C1); 133.08 (d, ^1^*J*_CP_ = 46, *C_ipso_*-PPh_3_); 132.30 (*C_para_*-PPh_3_); 130.43 (C5); 130.24 (C6); 130.11 (d,^3^*J*_CP_ = 10, *C_meta_-*PPh_3_); 97.16 (C2); 86.17 (η^5^-C_5_H_5_). ^31^P NMR [(CD_3_)_2_CO-d_6_, H_3_PO_4_, δ/ppm]: 66.51. ^31^P NMR [(CD_3_)_2_CO-d_6_, H_3_PO_4_, δ/ppm]: 66.39. 

#### 3.1.10. [Fe(η^5^-C_5_H_5_)(CO)(PPh_3_)(cis-NCCH=CHPhCl)][CF_3_SO_3_]

^1^H NMR [(CD_3_)_2_CO-d_6_, Me_4_Si, δ/ppm]: ^1^H NMR [(CD_3_)_2_CO-d_6_, Me_4_Si, δ/ppm]: 7.72–7.44 (comp, 19H, PPh_3_+H5+H6); 7.38 (d, *J*_HH_ = 16, H3); 5.76 (d, *J*_HH_ = 16, H2); 5.19 (s, 5, (η^5^-C_5_H_5_)). ^13^C NMR [(CD_3_)_2_CO-d_6_, Me_4_Si, δ/ppm]: 217.24 (d, *J*_CP_ = 26, C≡O); 151.72 (C3); 137.56 (C7); 135.44 (C4); 134.06 (d, ^2^*J*_CP_ = 10, *C_ortho_*-PPh_3_); 133.08 (d, ^1^*J*_CP_ = 46, *C_ipso_*-PPh_3_); 132.84 (d, ^3^*J*_CP_ = 26, C1); 132.30 (*C_para_*-PPh_3_); 131.06 (C5); 130.15 (d,^3^*J*_CP_ = 10, *C_meta_-*PPh_3_); 129.99 (C6); 96.72 (C2); 86.17 (η^5^-C_5_H_5_). ^31^P NMR [(CD_3_)_2_CO-d_6_, H_3_PO_4_, δ/ppm]: 66.51. ^31^P NMR [(CD_3_)_2_CO-d_6_, H_3_PO_4_, δ/ppm]: 65.38.

### 3.2. X-Ray Crystal Structure Determination 

Three-dimensional X-ray data for [Fe(*η*^5^-Cp)(CO)(NCPh)(PPh_3_)][CF_3_SO_3_] **1**, [Fe(*η*^5^-Cp)(CO)(*p*-NCPhNH_2_)(PPh_3_)][CF_3_SO_3_] **4** and [Fe(*η*^5^-Cp)(CO)(*p*-NCPhBr)(PPh_3_)][CF_3_SO_3_] **5** were collected on a Bruker SMART Apex CCD diffractometer (Bruker España, Rivas-Vaciamadrid, Madrid) at 100(2) K, using a graphite monochromator and Mo−*K_α_* radiation (*λ* = 0.71073 Å) by the *ϕ-ω* scan method. The reflections were measured from a hemisphere of data collected of frames each covering 0.3 degrees in *ω*. A total of 49157 for **1**, 105999 for **4** and 101816 for **5** reflections were measured, all of which were corrected for Lorentz and polarization effects and for absorption by semi-empirical methods that were based on symmetry-equivalent and repeated reflections. Of the total, 3956 for **1**, 5839 for **4**, and 5616 for **5** independent reflections exceeded the significance level |F|/σ(|F|) > 4.0. After data collection, in each case, a multi-scan absorption correction (SADABS) [29] was applied, and the structures were solved by direct methods and refined by full matrix least-squares on *F*^2^ data while using the SHELX suite of programs [30]. The structures were solved by direct methods and refined by full-matrix least-squares methods on F^2^. The non-hydrogen atoms were refined with anisotropic thermal parameters in all cases. The hydrogen atoms were located in difference Fourier map and left to refine freely, except to for C(2) and C(3) in complex **5**, which were included in calculation positions and refined in the riding mode. A final difference Fourier map showed no residual density outside: 0.631 and −0.461 e.Å^−3^ for **1**, 0.378 and −0.365 e.Å^−3^ for **4** and 1.279 and −0.543 e.Å^−3^ for **5**. A weighting scheme w = 1/[σ^2^(F_o_^2^) + (0.034700 P)^2^ + 1.212300 P] for **1,** 1/[σ^2^(Fo^2^) + (0.027800 P)^2^ + 2.298100 P] for **4** and 1/[σ^2^(Fo^2^) + (0.033400 P)^2^ + 8.005600 P] for **5**, where P = (|F_o_|^2^ + 2|F_c_|^2^)/3, were used in the latter stages of refinement. CCDC No. 1985552-1985554 contain the supplementary crystallographic data for **1**, **4,** and **5**. These data can be obtained free of charge via http://www.ccdc.cam.ac.uk/conts/retrieving.html, or from the Cambridge Crystallographic Data Centre, 12 Union Road, Cambridge CB2 1EZ, UK; fax: (+44) 1223-336-033; or e-mail: deposit@ccdc.cam.ac.uk. Table S4 illustrates crystal data and details of the data collection and refinement for the new compounds.

### 3.3. TD-DFT Calculations

All of DFT calculations were performed with Gaussian09 [31] using the PBE0 functional [32]. For iron and phosphorus, the LANL2TZ(f) and LANL08(d) basis sets with the associated effective core potential were used, respectively [33,34]. For the other elements, a standard 6-311G** was employed. After geometry optimization, TD-DFT calculations were performed, typically requesting the lowest allowed 20 excitations. The effect of the solvent (dichloromethane) was accounted for using the SMD solvation model, as implemented in Gaussian 09 [35]. The electron density difference maps (EDDMs) were obtained while using the GaussSum package [36].

### 3.4. Biological Studies

#### 3.4.1. Cell Lines and Culture Conditions

The colorectal cancer-derived cell line SW480 was obtained from American Type Culture Collection (ATCC) (Manassas, VI, USA), and the noncancerous NCM460 cell line that was derived from normal colon epithelial mucosa was obtained from INCELL’s (San Antonio, TX, USA) [37]. Both cell lines were maintained at 37 °C under a humidified atmosphere containing 5% CO_2_. The SW480 and NCM460 cells were grown in Roswell Park Memorial Institute (RPMI) (Biowest, Nuaillé, France), supplemented with 10% FBS and 1% penicillin/streptomycin. The cells were subcultured once a week when 80% of confluence was reached and, when necessary, seeded in sterile test plates for the assays.

The MDA-MB-231 cells were grown at 37 °C in 5% CO_2_ in Dulbecco’s modified Eagles’s medium (DMEM high glucose) (Capricorn Scientific) supplemented with 10% fetal bovine serum (Capricorn Scientific). All of the cells were adherent in monolayers and, upon confluence, were washed with phosphate buffer saline (PBS) 1× and harvested by digestion with trypsin 0.05% (*v*/*v*). Trypsin was inactivated by adding fresh complete culture media to the culture flask. The cells were then suspended and transferred into new, sterile, culture flasks, or seeded in sterile test plates for the different assays. All of the cells were manipulated under aseptic conditions in a flow chamber.

#### 3.4.2. Compounds Dilution and Storage

The compounds were dissolved in DMSO. The aliquots were prepared in sterile conditions and stored at −20 °C, protected from light, and after thawing were discarded. Cisplatin was used as a positive control and dissolved in a sterile filtered solution of sodium chloride (NaCl) 0.9% (*w*/*v*) in deionized water, stored at −20 °C, and protected from light.

#### 3.4.3. Sulforhodamine B (SRB) Assay

The SW480 and NCM460 cells were seeded at a concentration of 1 × 10^5^ cells/ml and 2 × 10^5^ cells/ml, respectively, in 24-well test plates. After 24 h of seeding, the cells were incubated with different concentrations of the compounds, during 48 h. For each cell line and compound, we performed two negative controls, a control (1), in which the cells were only incubated with growth medium and another DMSO control (2), in which the cells were exposed to the concentration of DMSO, in which the highest concentration of the compound was dissolved (maximum of 0.1% of DMSO per well (*v*/*v*)), to discard any influence of the DMSO in the results. After 48 h of treatment, cells were fixed in ice-cold methanol containing 1% acetic acid for at least 90 min, at −20 °C. The fixing solution was then removed, and the plate was left air-dry at room temperature. Fixed cells were incubated with 0.5% (*w*/*v*) SRB dissolved in 1% acetic acid for 90 min at 37 °C protected from light. After washing with 1% acetic acid and air-drying at room temperature, SRB was solubilized with 10 mM Tris pH10. Absorbance was read at 540 nm in a microplate reader (SpectraMax 340PC Molecular Devices, San José, CA, USA). The results were expressed relative to the negative control 1, which was considered as 100% of cell growth. The results were obtained from at least three independent experiments, and each experiment was done in triplicate. The IC_50_ values were estimated using GraphPad Prism 6 software, while applying a sigmoidal dose vs response (variable slope) non-linear regression (n = 3). To determine the cytotoxic selectivity of the substances tested, the selectivity index (SI) was calculated according to the following equation [38]:

SI = IC_50_ (normal cell line)/IC_50_ (cancer cell line) (1)

#### 3.4.4. MTT Assay

The cytotoxicity of the ligands and complexes against the MDA-MB-231 cells was assessed while using the colorimetric assay MTT (3-(4,5-2-yl)-2,5-ditetrazolium bromide), which measures the conversion of the yellow tetrazolium into purple formazan by mitochondrial redox activity in living cells. For this purpose, cells (5 × 10^3^ in 200 µL of medium) were seeded into 96-well plates and incubated in a 5% CO_2_ incubator at 37 °C. The cells settled for 24 h followed by the addition of a dilution series of the ligands (1–100 µM) and complexes (2–0.01 µM) in medium (200 µL). DMSO did not exceed 1%, even for the highest concentration and was without cytotoxic effect. After 48 h incubation, the treatment solutions were removed by aspiration and MTT solution (200 µL, 0.5 mg/mL in PBS) was added to each well. After 3–4 h at 37 °C/5% CO_2_, the solution was removed, and the purple formazan crystals that formed inside the cells were dissolved in DMSO (200 µL) by thorough shaking. The cellular viability was evaluated while measuring the absorbance at 570 nm by using a microplate spectrophotometer.

#### 3.4.5. Colony Formation Assay

The SW480 cell line was seeded, at a concentration of 750 cells/ml, in six-well plates. After 24 h of seeding, the cells were treated with ¼ IC_50_ and IC_50_ values of iron and cisplatin compounds. After 48 h, the cells were washed with PBS and the medium was replaced with fresh medium. The negative control cells were treated with DMSO 0.1%. Eight days later, the cells were washed with PBS and fixed with glutaraldehyde 6% (*v*/*v*) and crystal violet 0.5% (*w*/*v*) for three hours. Subsequently, the cells were washed with fresh water and the plate was left air-dry. Colonies were counted using ImageJ 1.50i software. The results represent mean ± S.D. of at least three independent experiments. Statistical analysis was performed by one-way ANOVA with Dunnett’s multiple comparisons test. ** *P* ≤ 0.01; **** *P* ≤ 0.0001 as compared with the negative control.

#### 3.4.6. Annexin V/Propidium Iodide Assay

The colorectal cancer-derived cell line SW480 was seeded, in six-well plates, at a concentration of 1 × 10^5^ cells/ml. Twenty-four hours after seeding, the cells were exposed to the IC_50_ and 2 × IC_50_ values of iron compounds and cisplatin. The negative control cells were treated with DMSO 0.1%. After 48 h, both floating and attached cells were collected and washed with PBS. The cell suspension collected was centrifuged at 2500 rpm for 10 min and the pellet resuspended in binding buffer. Afterwards, 5 µL of Annexin V (AV) and 5 µL of Propidium Iodide (PI) at 50 µg/mL were added to each sample and acquisition was performed using CytoFLEX Cytometer (Beckman Coulter, Brea, CA, USA). The values represent mean ± S.D. of at least three independent experiments. Statistical analysis was performed by two-way ANOVA with Dunnett’s multiple comparisons test. * *P* ≤ 0.05; ** *P* ≤ 0.01; *** *P* ≤ 0.001; **** *P* ≤ 0.0001 when compared with negative control.

#### 3.4.7. F-Actin Staining Assay with Phalloidin 568

SW480 cell line was seeded at a concentration of 1 × 10^5^ cells/ml, in 12-well plates with one coverslip per well. After 24 h, the cells were exposed to the IC_50_ values of iron compounds and cisplatin, and the cells of negative control were treated with DMSO 0.1%. After 48 hours of incubation, the cells were washed twice with PBS and fixed with paraformaldehyde 4% (*w*/*v*) for 10 minutes. After fixation, cells were incubated with NH_4_Cl 50 mM and washed twice with PBS, for five minutes. The cells were permeabilized using Triton X-100 0.2%, for five minutes, and blocked with PBS–BSA 3% for 20 minutes. Subsequently, cells were incubated with Alexa Fluor™ 568 Phalloidin (ThermoFisher Scientific®, Waltham, MA, USA), diluted in PBS (1:40), for one hour in the dark, and washed twice with PBS. To finalize, coverslips were mounted using 5 μL of VECTASHIELD Antifade Mounting Medium with DAPI (Vector Laboratories^®^, Peterborough, UK) in microscope slides. The results were obtained from at least three independent experiments. Representative images were obtained in a fluorescence microscope (Olympus motorized BX63F Upright Microscope) (Olympus©, Tokyo, Japan) at a magnification of 600×. 

## 4. Conclusions

In the continuation of our work on the ‘FeCp’ scaffold, a new family of cationic complexes with the general formula [Fe(η^5^-C_5_H_5_)(CO)(PPh_3_)(NCR)]^+^ has been synthesized. All of the compounds were fully characterized by the usual spectroscopic techniques. X-ray studies for compounds **1**, **4**, and **5** indicate that they crystalize in the monoclinic space group P2_1_/c and adopt the expected piano-stool geometry. The DFT studies allowed for a clearer interpretation of the UV-Vis. data, showing that the low-lying energy transitions are highly mixed, with contributions from d-d, iron to phosphane, and iron-nitrile ligand to phosphane charge transfers. All of the compounds are cytotoxic in breast MDA-MB-231 and colorectal SW480 cancer cells with IC_50_ at a low micromolar range. These compounds cause cell death by apoptosis, inhibiting the formation of colonies and affecting cell cytoskeleton organization. Overall, the biological data revealed that the substituents at the nitrile ligands induce different biological responses, particularly the hydroxylated compounds (**2** and **3**) seem to present an alternative mode of action interfering less with the cytoskeleton of cells.

## Figures and Tables

**Table 1:** Selected ^1^H NMR chemical shifts (ppm). The spectra were obtained in acetone-d_6_. Values in brackets represent the differences on H_3_ between the coordinated and free nitrile.

Complex	(η^5^-C_5_H_5_)	H3
[Fe(η^5^-C_5_H_5_)(CO)(PPh_3_)I]	4.57	-
**1**	5.21 (+0.64)	7.37 (−0.37)
**2**	5.17 (+0.60)	7.19 (−0.43)
**3**	5.20 (+0.63)	7.48 (−0.26)
**4**	5.13 (+0.56)	6.97 (−0.42)
**5**	5.21 (+0.64)	7.31 (−0.45)

**Table 2:** Characteristic stretching frequencies (cm^−1^). Values in brackets represent the differences between the υ(N≡C) of coordinated and free nitrile or between the υ(CO) of [Fe(η^5^-C_5_H_5_)(CO)(PPh_3_)I] and compounds **1**–**6**.

Complex	υ_N≡C_	υ_CO_
[Fe(η^5^-C_5_H_5_)(CO)(PPh_3_)I]	-	1935
**1**	2247 (+19)	1977 (+42)
**2**	2249 (+16)	1986 (+51)
**3**	2250 (+23)	1984 (+49)
**4**	2237 (+23)	1980 (+45)
**5**	2245 (+22)	1996 (+61)
**6**	2241 (+25)	1956 (+21)

**Table 3:** IC_50_ values determined at 48 h incubation of iron compounds (**1**–**6**) against MDA-MB-231 breast cancer cells, SW480 colorectal cancer cells and noncancerous cell line NCM460, derived from normal colon epithelial cells.

	IC_50_ (μM)			
Compounds	MDA-MB-231	SW480	NCM460	Selectivity Index
**1**	0.45 ± 0.02	1.5 ± 0.2	3.8 ± 0.6	2.5
**2**	0.71 ± 0.05	3.0 ± 0.3	7.8 ± 1.2	2.6
**3**	0.37 ± 0.04	3.0 ± 0.2	11.7 ± 1.8	3.9
**4**	0.19 ± 0.01	1.0 ± 0.1	2.4 ± 0.5	2.4
**5**	0.91 ± 0.05	1.5 ± 0.1	3.0 ± 0.4	2.0
**6**	0.39 ± 0.02	1.0 ± 0.1	1.8 ± 0.1	1.8

## Supplementary Materials

The following is available online, Figure S1—^1^H NMR spectrum of complexes 6, in acetone-d6, Table S1. Bond lengths [Å] and angles [°] for [Fe(η^5^-Cp)(CO)(PhCN)(PPh_3_)][CF_3_SO_3_] 1, [Fe(η^5^-Cp)(CO)(*p*-NCPhNH_2_)(PPh_3_)][CF_3_SO_3_] 4 and [Fe(η^5^-Cp)(CO)(*p*-NCPhBr)(PPh_3_)][CF_3_SO_3_] 5, Table S2. Relevant TD-DFT (PBE0) excitation energies (λ), oscillator strengths (f) and compositions (only those > 5% are shown), for complexes 1–6, compared with experimental data (λexp). Both calculated and experimental values were obtained in dichloromethane, Figure S2—UV-Vis spectra of complexes 1–6 in DMSO along the 24 h study, Figure S3—UV-Vis spectra of complexes 1–6 in DMSO/DMEM mixture along the 24 h study and its variation plot (%) (bottom), Figure S4. ‘FeCp’ compounds affect the colony formation ability of SW480 cell line. Analysis of the colony formation ability, after 48 h of incubation with 1/4 IC_50_ and IC_50_, in SW480 cell line. Representative images of colony formation assay in SW480 cell line, Figure S5. ‘FeCp’ compounds induce apoptosis colorectal cancer-derived cell line. Apoptotic cell death was analyzed by Annexin V fluorescein isothiocyanate (AV-FITC) and propidium iodide (PI) assay in SW480 cells, after incubation with IC_50_ and 2×IC_50_ concentrations for 48 h. Representative histograms of SW480 cell line double stained with AV and PI, Table S4. Crystal data and structure refinement for [Fe(η^5^-Cp)(CO)(PhCN)(PPh_3_)][CF_3_SO_3_] 1, [Fe(η^5^-Cp)(CO)(*p*-NCPhNH_2_)(PPh_3_)][CF_3_SO_3_] 4 and [Fe(η^5^-Cp)(CO)(*p*-NCPhBr)(PPh_3_)][CF_3_SO_3_] 5.
