# Supplementary material for: A New Family of Iron(II)-Cyclopentadienyl Compounds Shows Strong Activity against Colorectal and Triple Negative Breast Cancer Cells
Source: Molecules. 2020 Mar 30;25(7):1592. doi: 10.3390/molecules25071592 (PMC7180908; doi:10.3390/molecules25071592)
Supplement: Supplementary file 1 [file molecules-25-01592-s001.pdf]

Supporting Information

A New Family of Iron(II)-Cyclopentadienyl Compounds Shows Strong Activity Against Colorectal and Triple Negative Breast Cancer Cells

Adhan Pilon [1,2], Ana Rita Brás [1,3,4], Leonor Côrte-Real [1,2], Fernando Avecilla [5], Paulo J. Costa [6], Ana Preto [3,4], M. Helena Garcia [1,2,*] and Andreia Valente [1,2,*]

1. Centro de Química Estrutural, Faculdade de Ciências, Universidade de Lisboa, Campo Grande, 1749-016 Lisboa, Portugal; fc42762@alunos.fc.ul.pt (A.P.); pg31015@alunos.uminho.pt (A.R.B.); ldcortereal@fc.ul.pt (L.C.-R.)
2. Departamento de Química e Bioquímica, Faculdade de Ciências, Universidade de Lisboa, Campo Grande, 1749-016 Lisboa, Portugal
3. Centre of Molecular and Environmental Biology, Department of Biology, University of Minho, Campus de Gualtar, 4710-057 Braga, Portugal; apreto@bio.uminho.pt
4. Institute of Science and Innovation for Bio-Sustainability, University of Minho, Campus de Gualtar, Edifício 18, 4710-057 Braga, Portugal
5. Grupo Xenomar, Centro de Investigacións Científicas Avanzadas (CICA), Departamento de Química, Facultade de Ciencias, Universidade da Coruña, Campus de A Coruña, 15071 A Coruña, Spain; fernando.avecilla@udc.es
6. University of Lisboa, Faculty of Sciences, BioISI—Biosystems & Integrative Sciences Institute, Campo Grande, C8 bdg, 1749-016 Lisboa, Portugal; pjcosta@fc.ul.pt

* Correspondence: amvalente@fc.ul.pt (A.V.); mhgarcia@fc.ul.pt (M.H.G.); Tel.: +351-217500955 (A.V.)

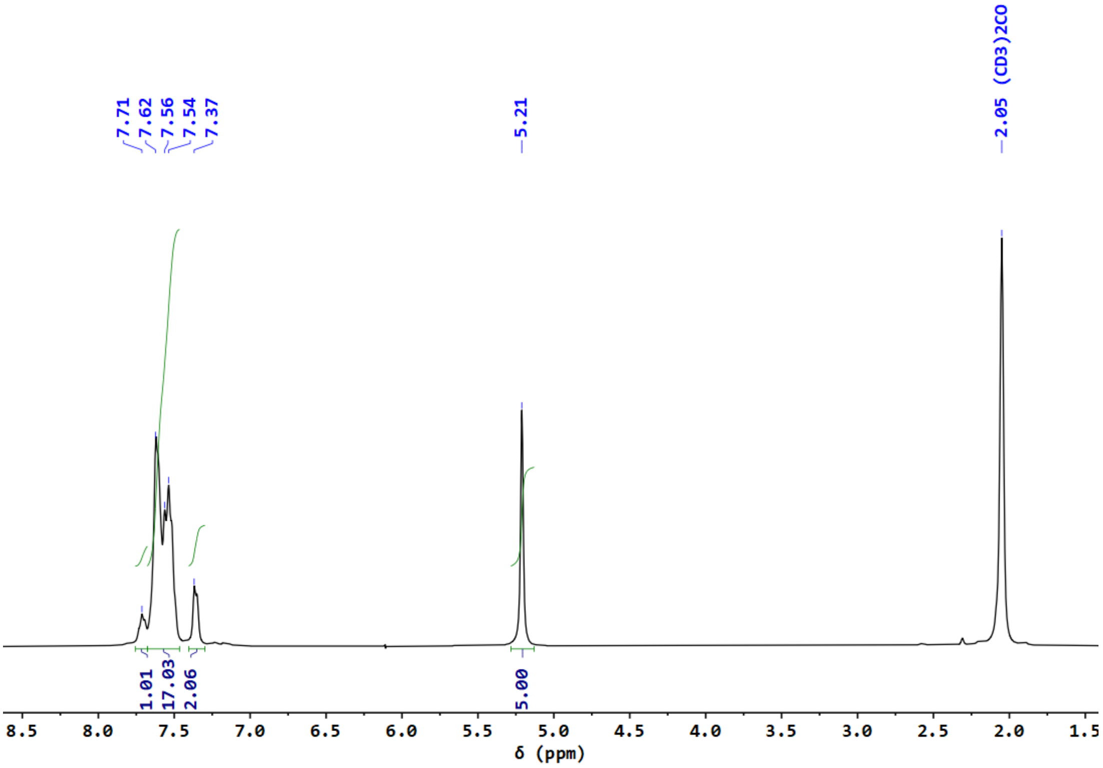

Figure S1 - ^{1}H NMR spectrum of complex **1**, in acetone-d_6

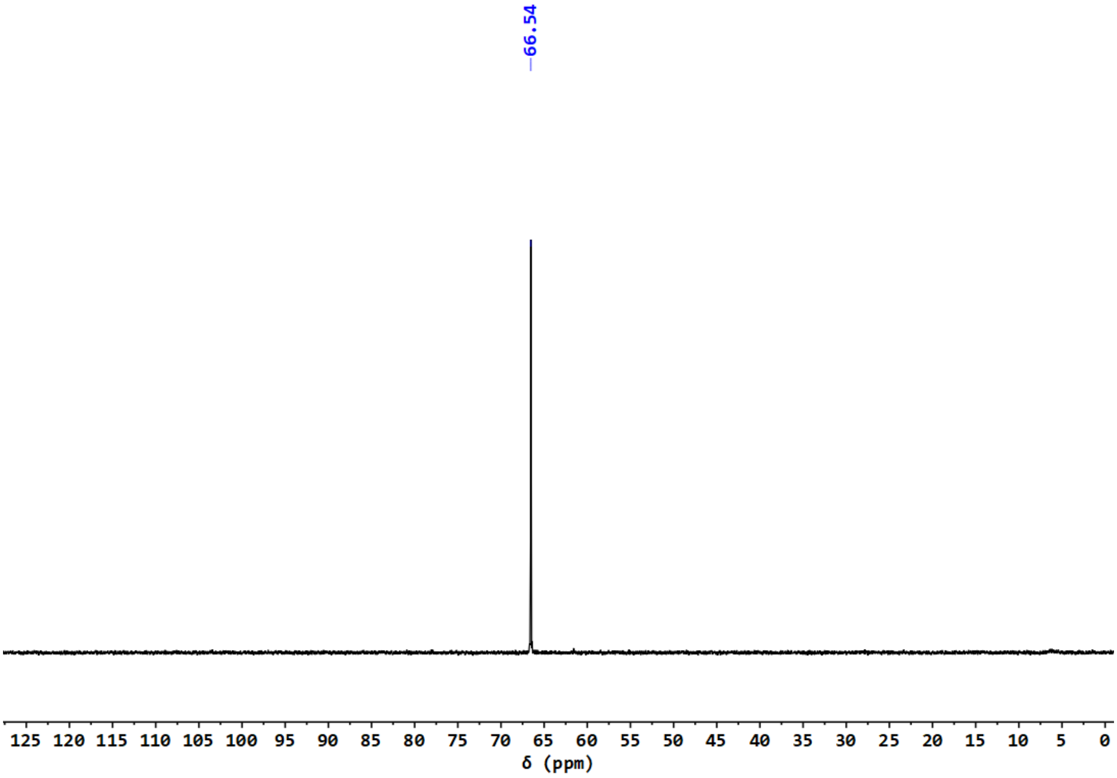

Figure S2 – $^{31}P\{^{1}H\}$ NMR spectrum of complex **1**, in acetone-d_6

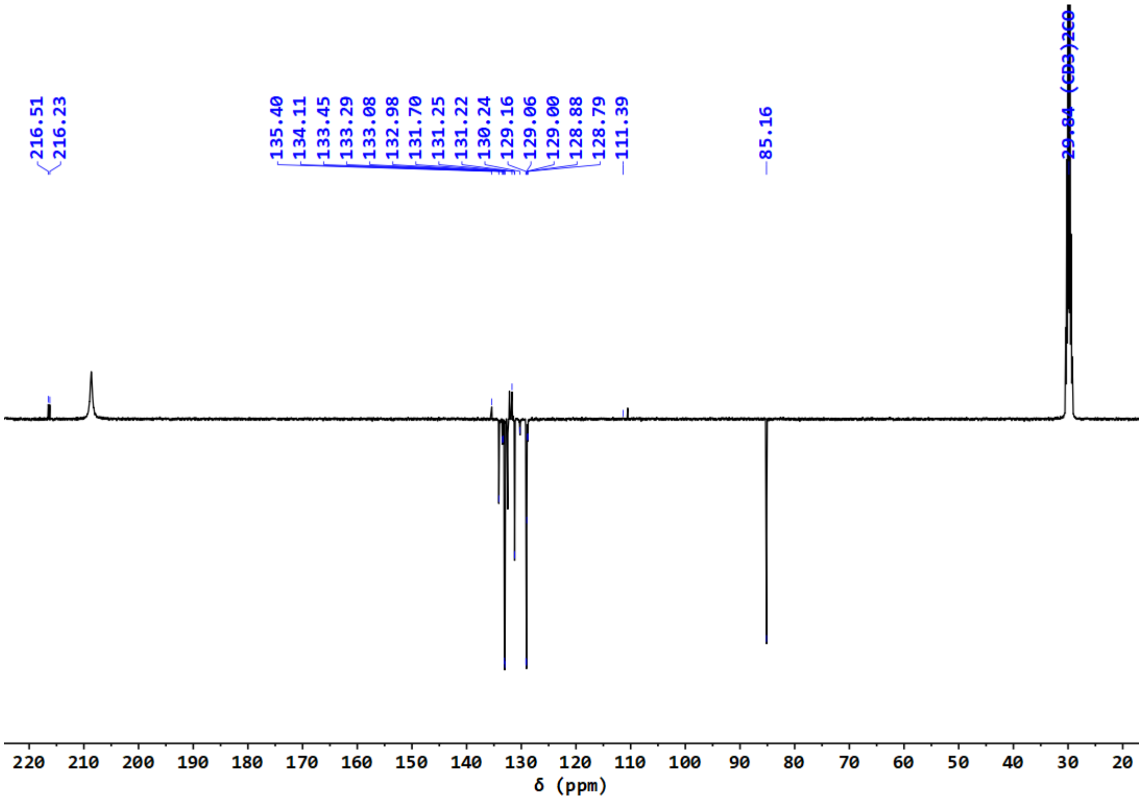

Figure S3 – $^{13}C\{^{1}H\}$-apt NMR spectrum of complex **1**, in acetone-d_6

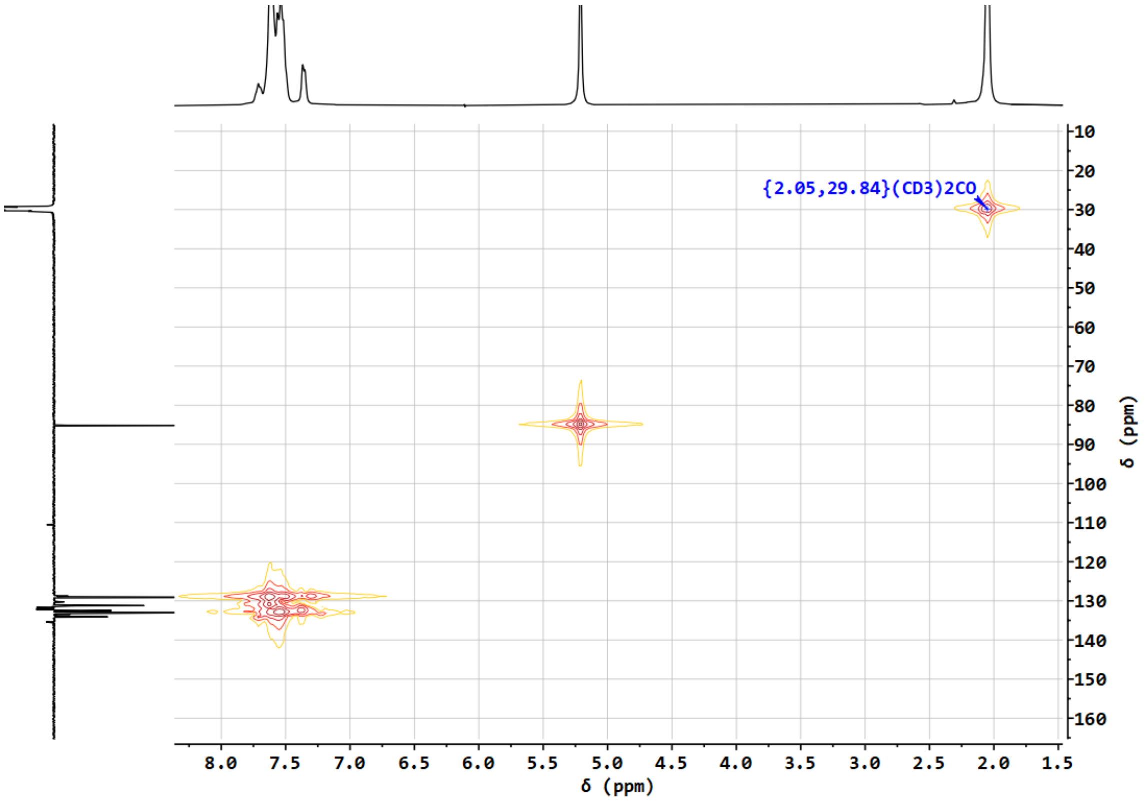

Figure S4 – HMQC spectrum of complex **1**, in acetone-d_6

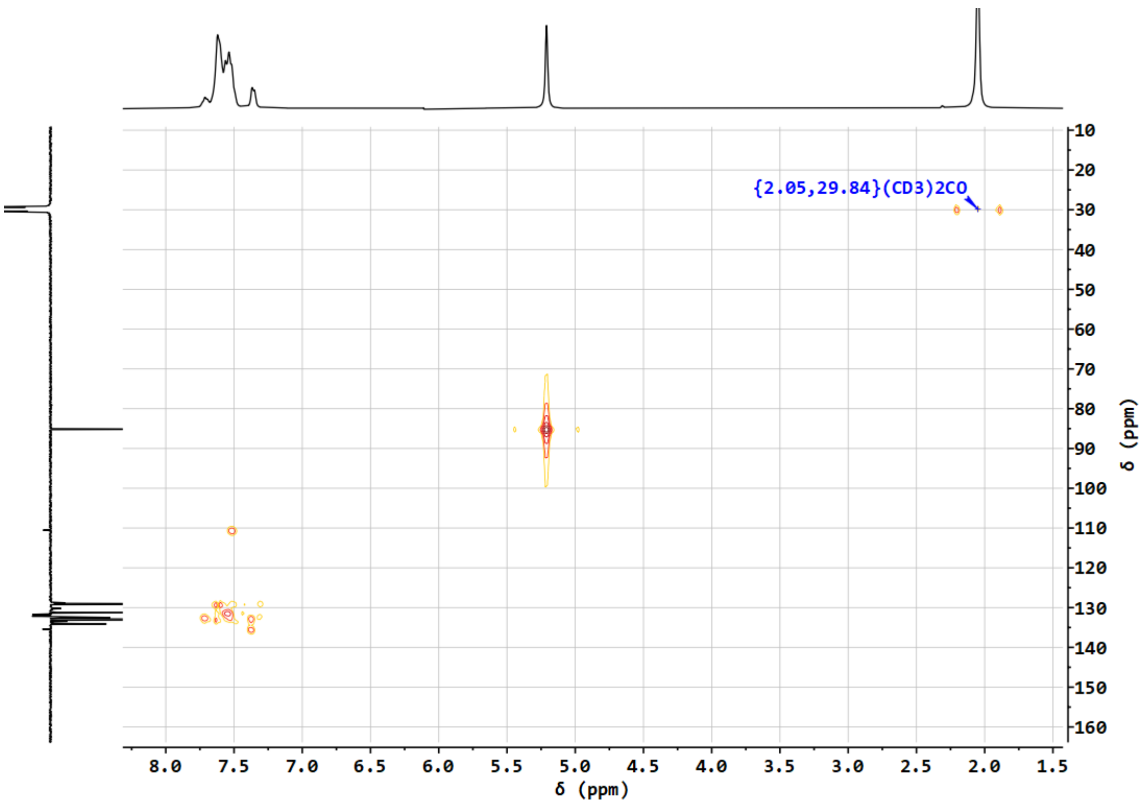

Figure S5 – HMBC spectrum of complex **1**, in acetone-d_6

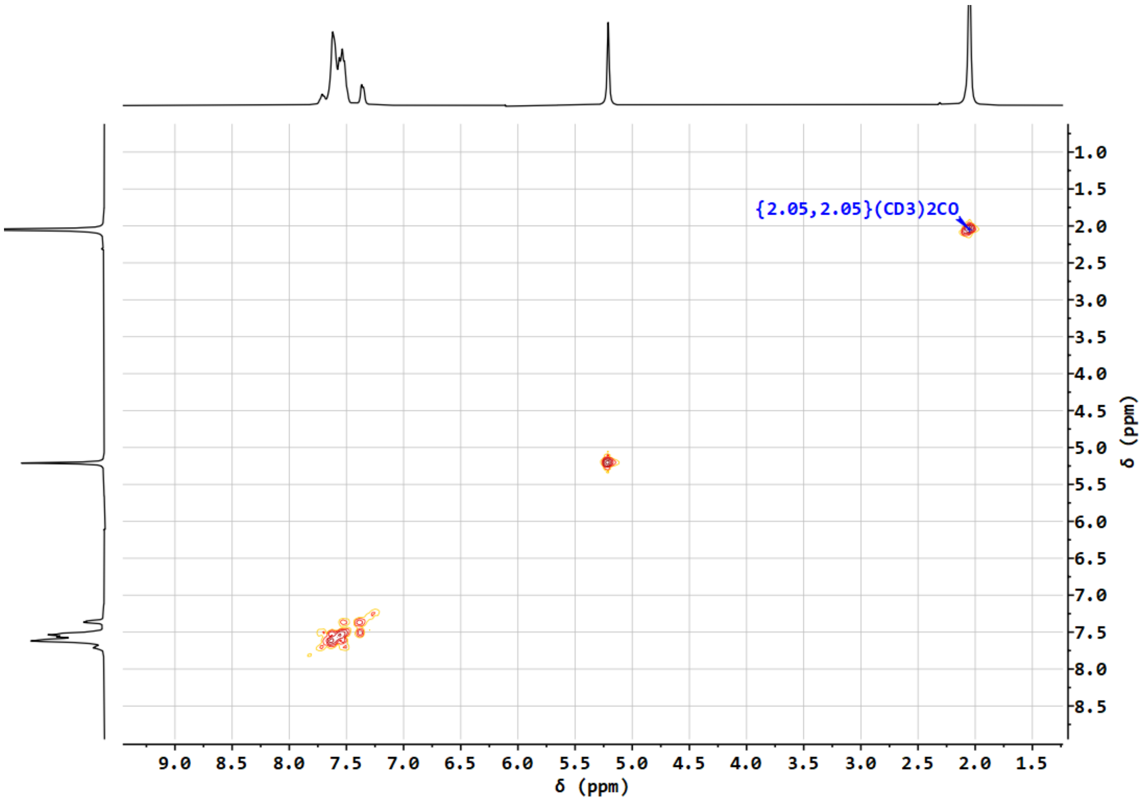

Figure S6 – COSY spectrum of complex **1**, in acetone-d_6

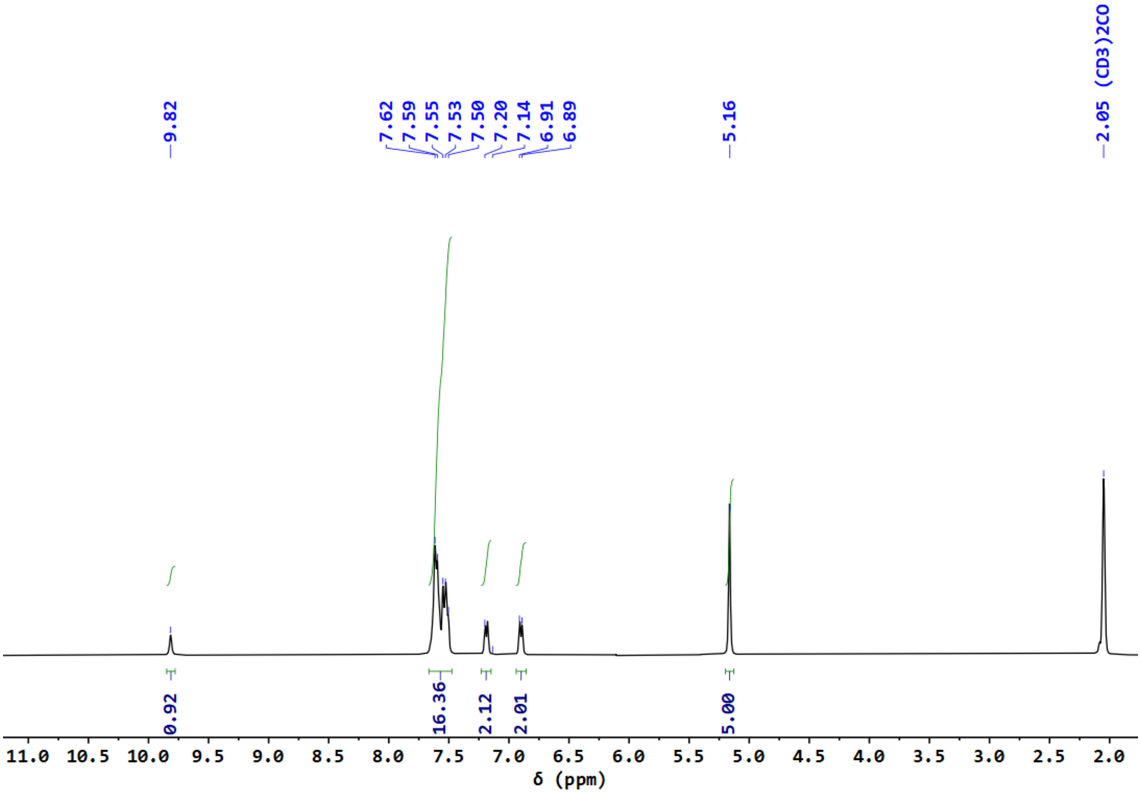

Figure S7 - ^{1}H NMR spectrum of complex **2**, in acetone-d_6

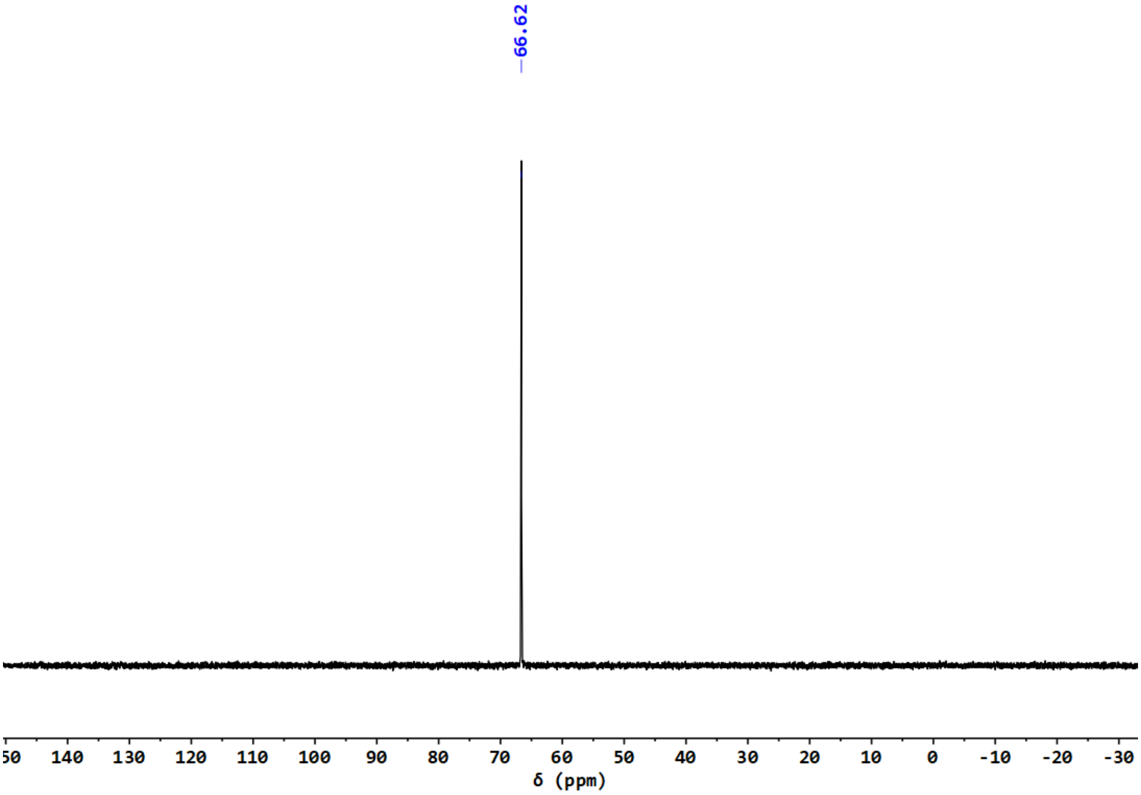

Figure S8 - $^{31}P\{^{1}H\}$ NMR spectrum of complex **2**, in acetone-d_6

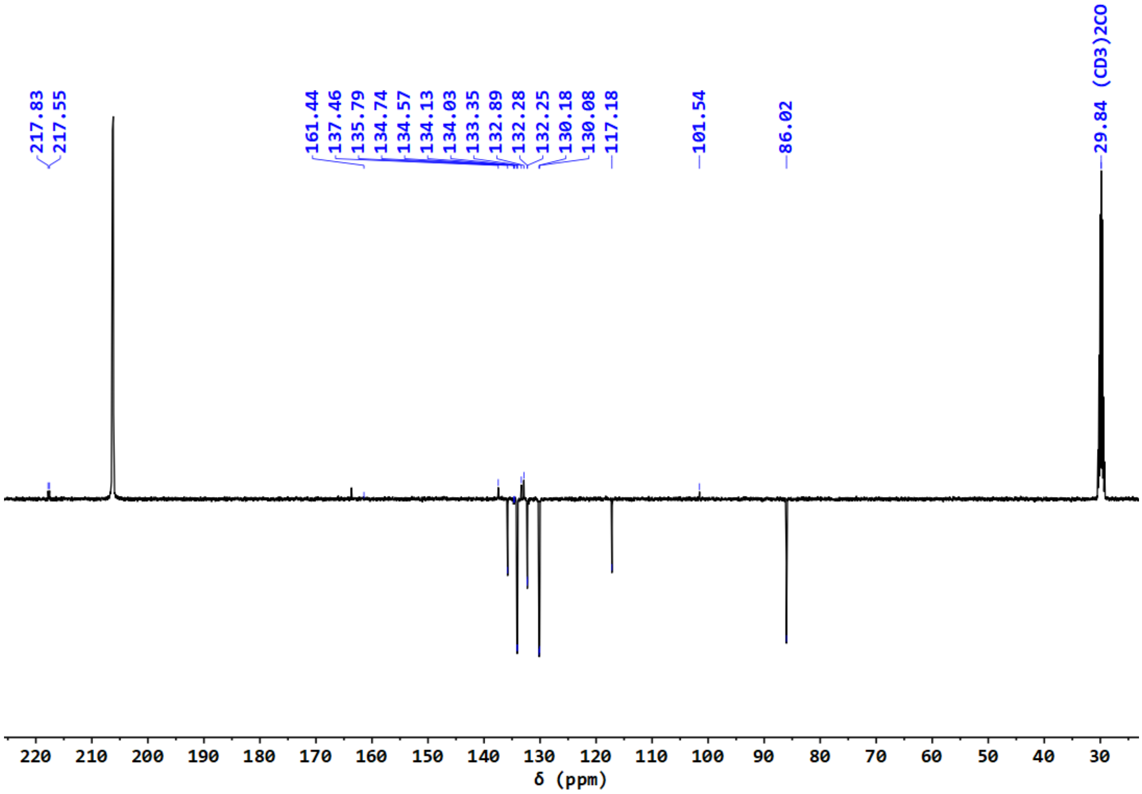

Figure S9 – $^{13}C\{^{1}H\}$-apt NMR spectrum of complex **2**, in acetone-d_6

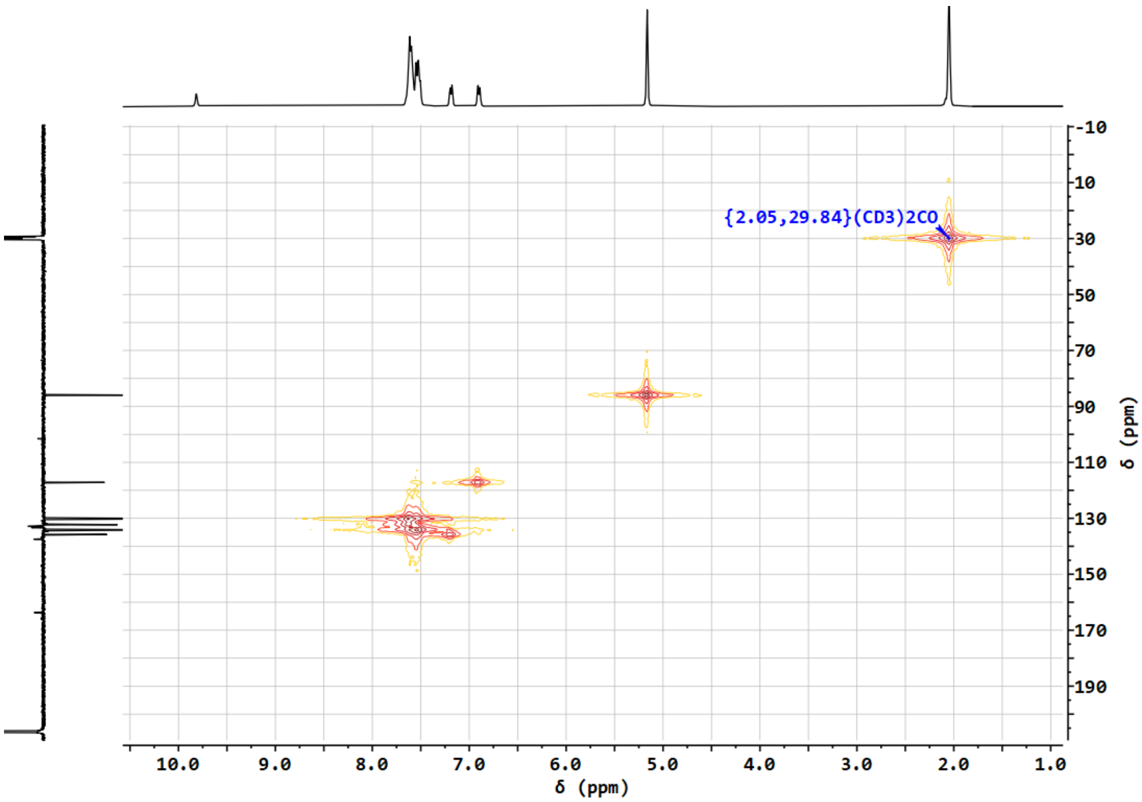

Figure S10 - HMQC spectrum of complex **2**, in acetone-d_6

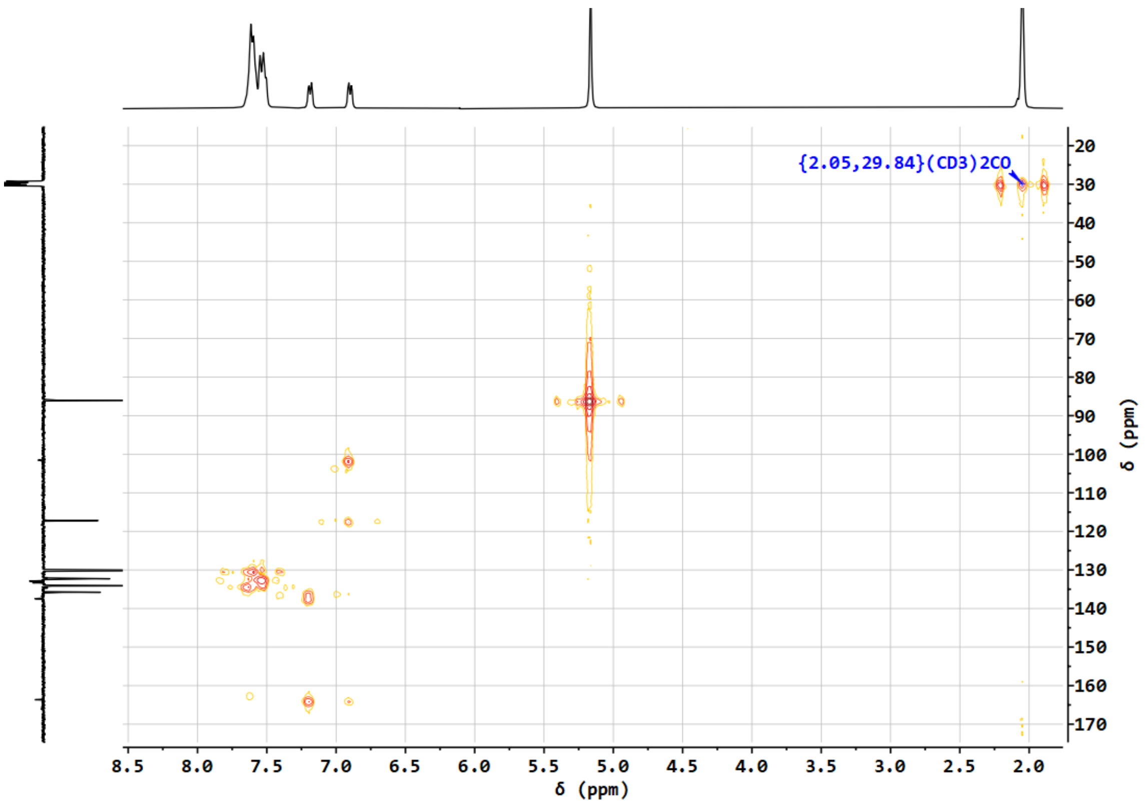

Figure S11 - HMBC spectrum of complex **2**, in acetone-d_6

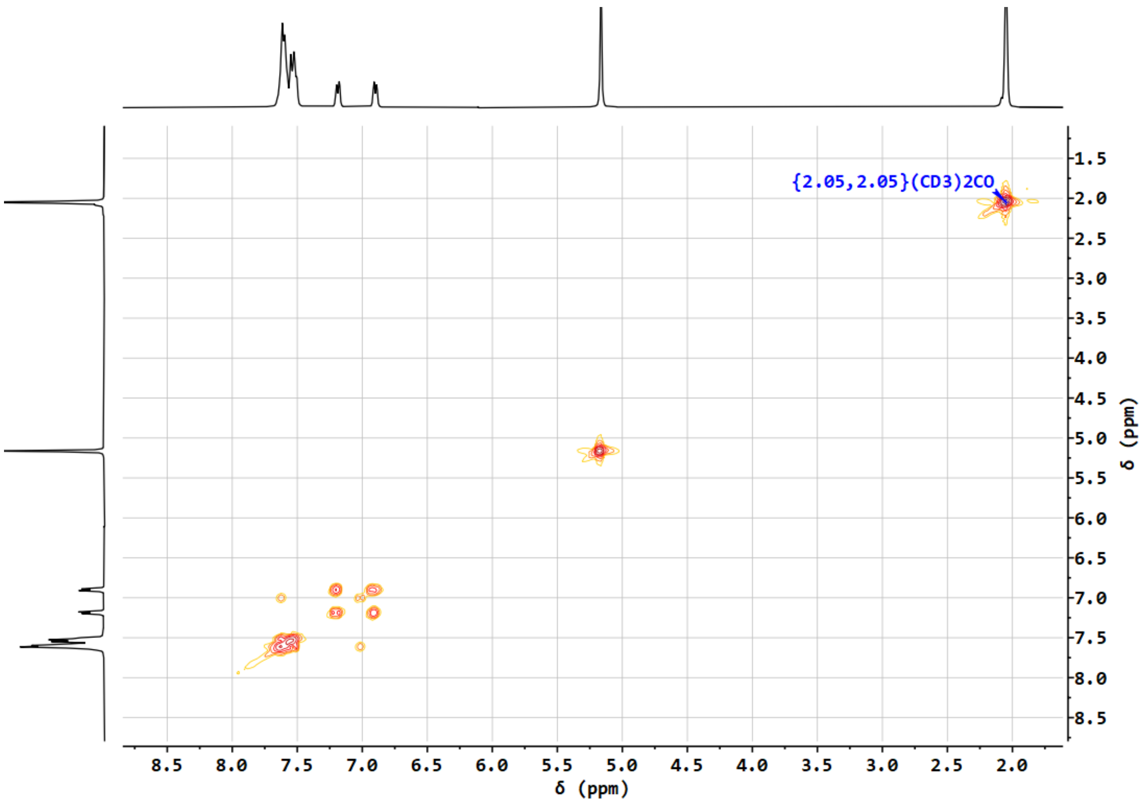

Figure S12 - COSY spectrum of complex **2**, in acetone-d_6

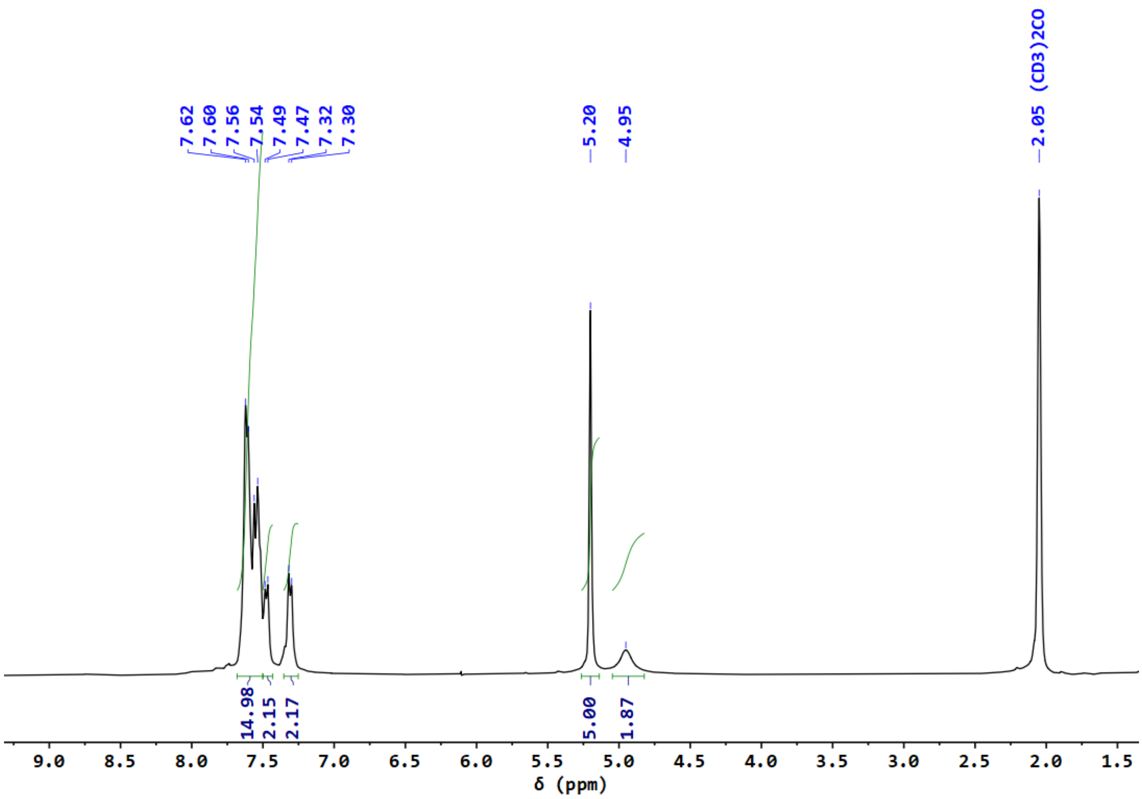

Figure S13 - ^{1}H NMR spectrum of complex **3**, in acetone-d_6

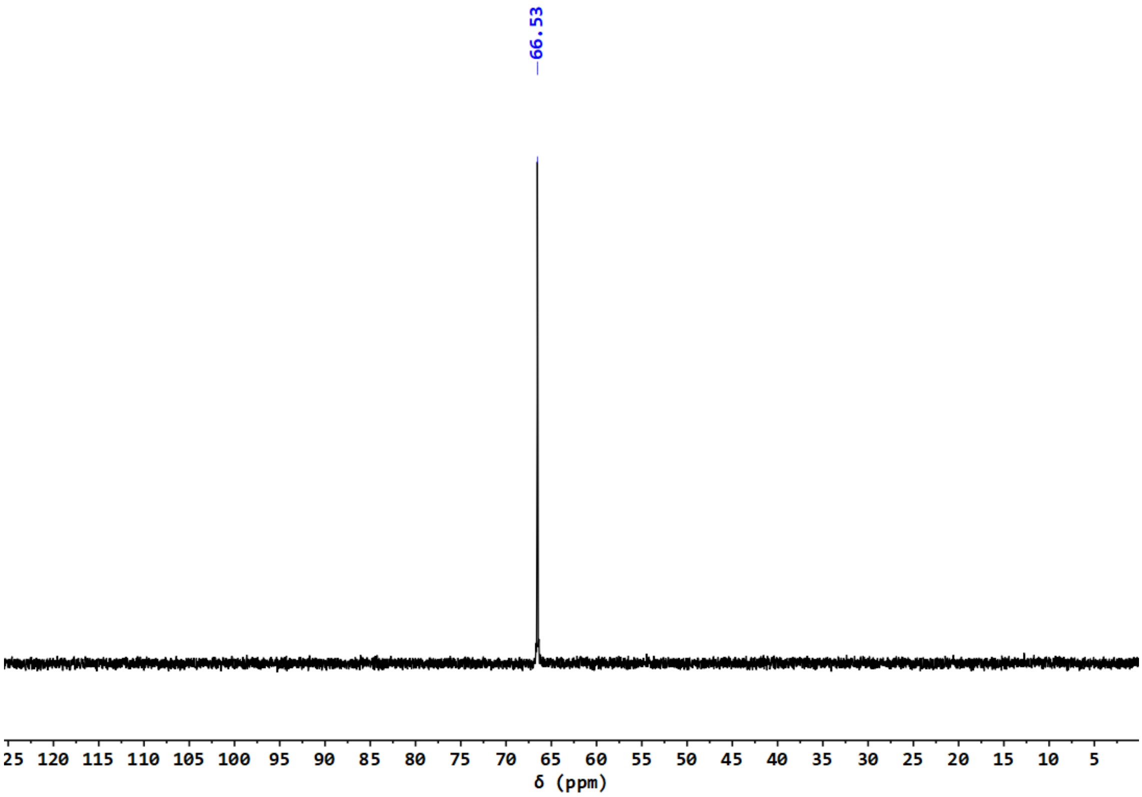

Figure S14 – ^{31}P{^{1}H} NMR spectrum of complex **3**, in acetone-d_6

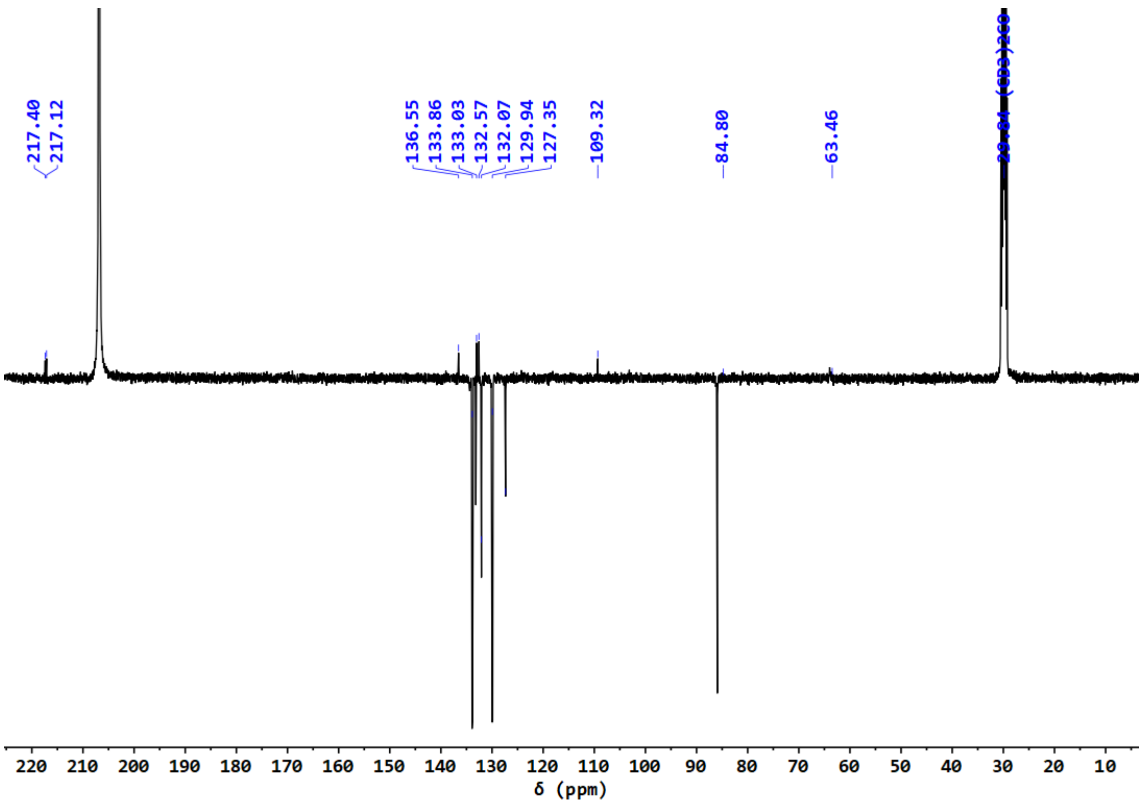

Figure S15 – $^{13}C\{^{1}H\}$-apt NMR spectrum of complex **3**, in acetone-d$_6$

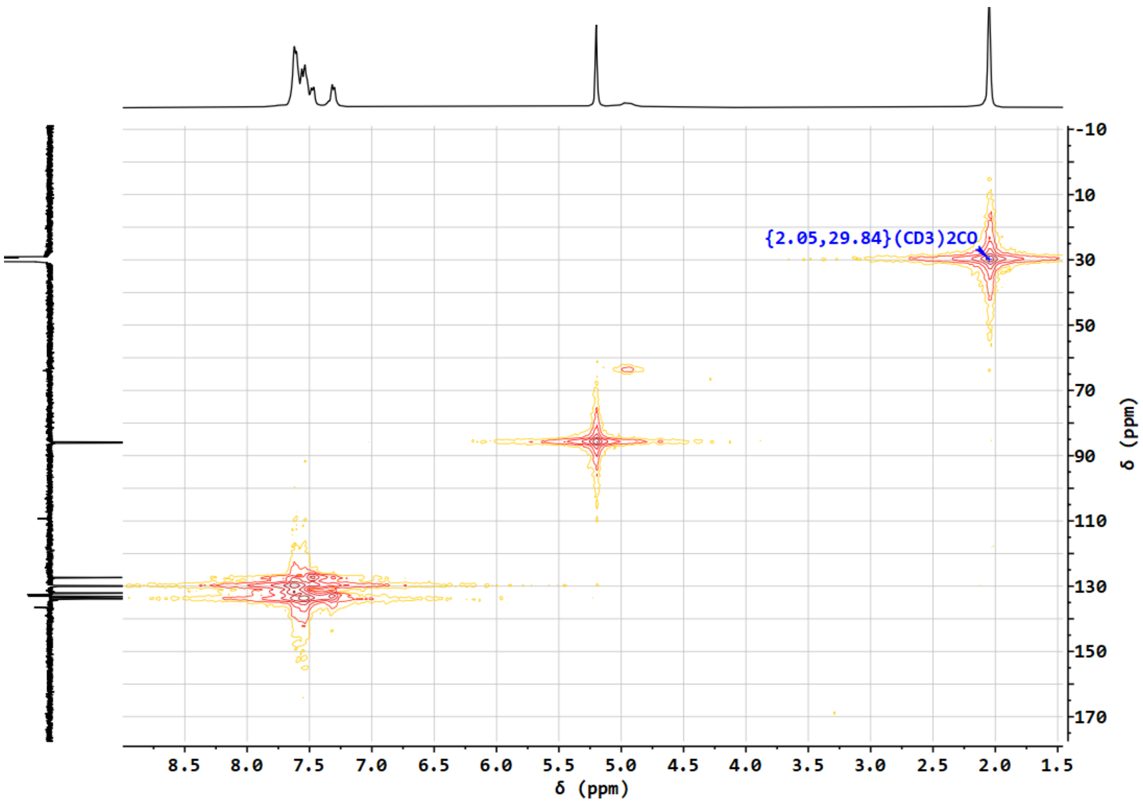

Figure S16 – HMQC spectrum of complex **3**, in acetone-d$_6$

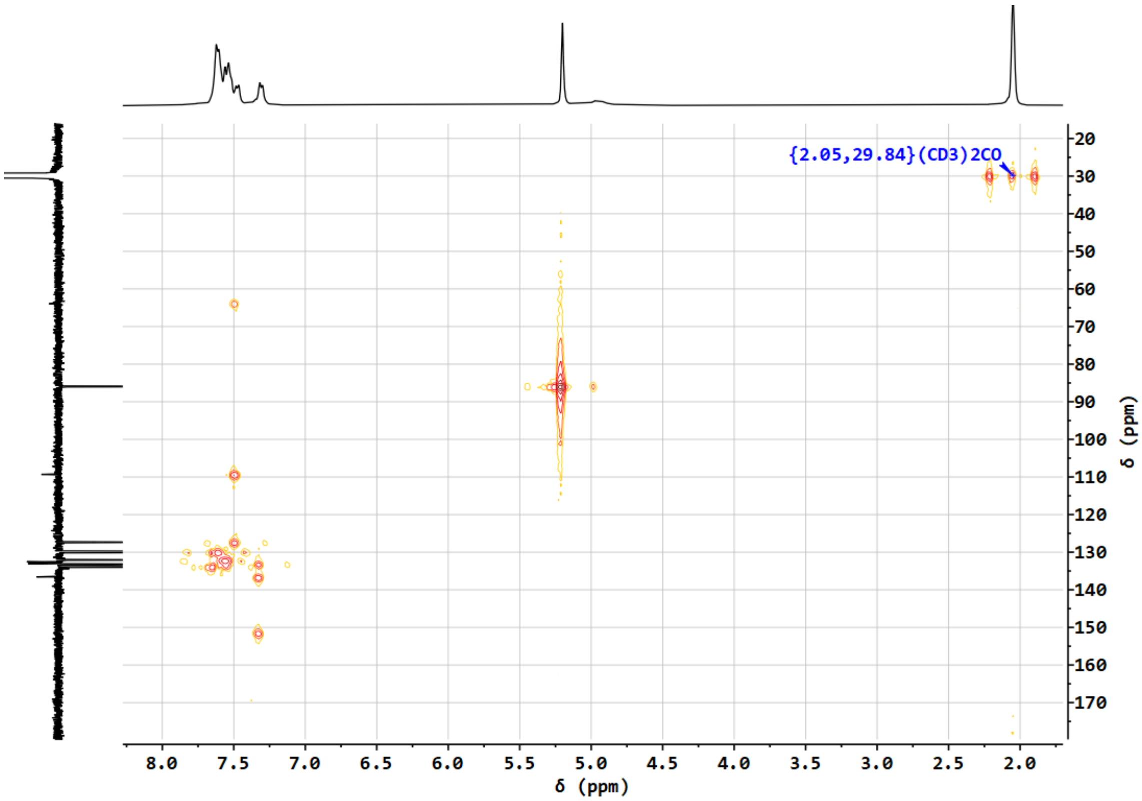

Figure S17 – HMBC spectrum of complex **3**, in acetone-d_6

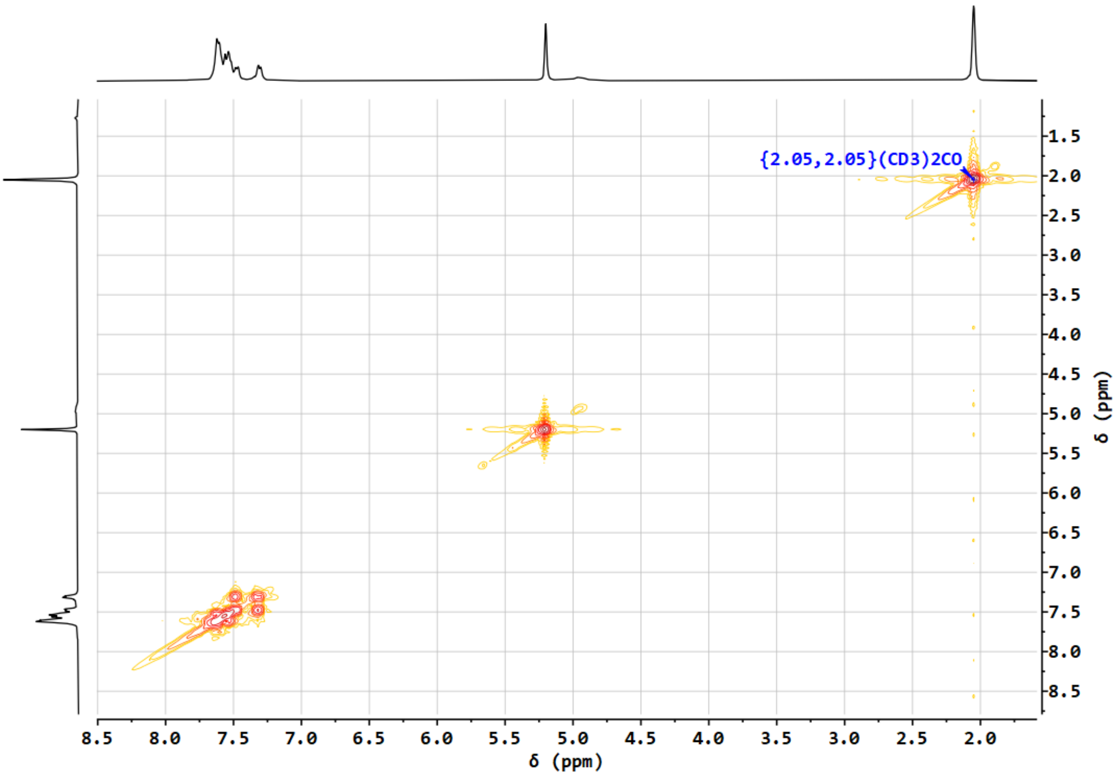

Figure S18 – COSY spectrum of complex **3**, in acetone-d_6

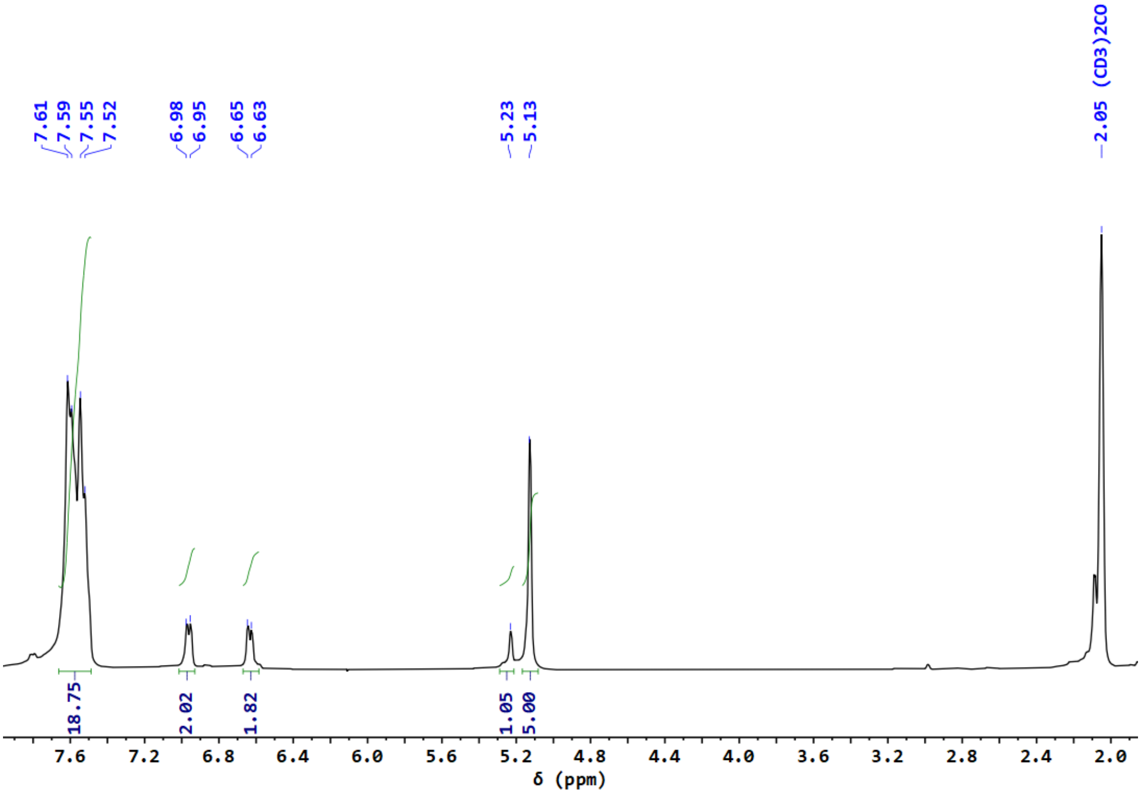

Figure S19 - ^{1}H NMR spectrum of complex **4**, in acetone-d_6

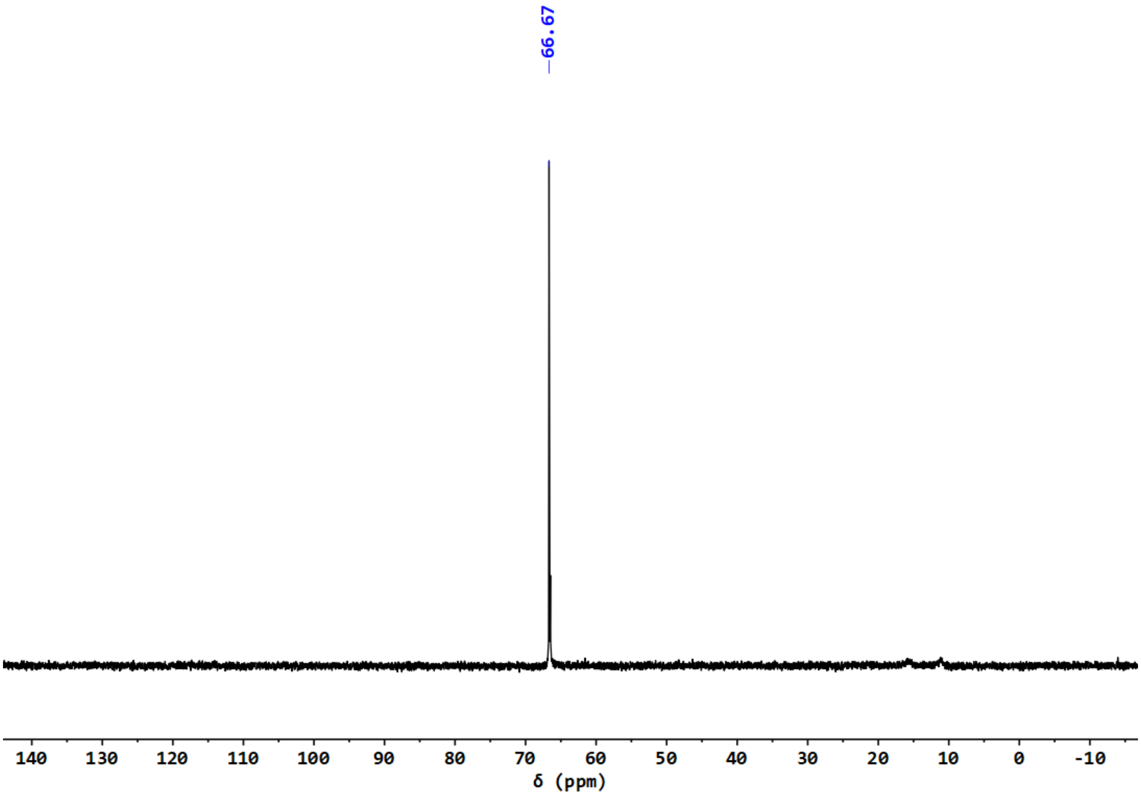

Figure S20 – ^{31}P{^{1}H} NMR spectrum of complex **4**, in acetone-d_6

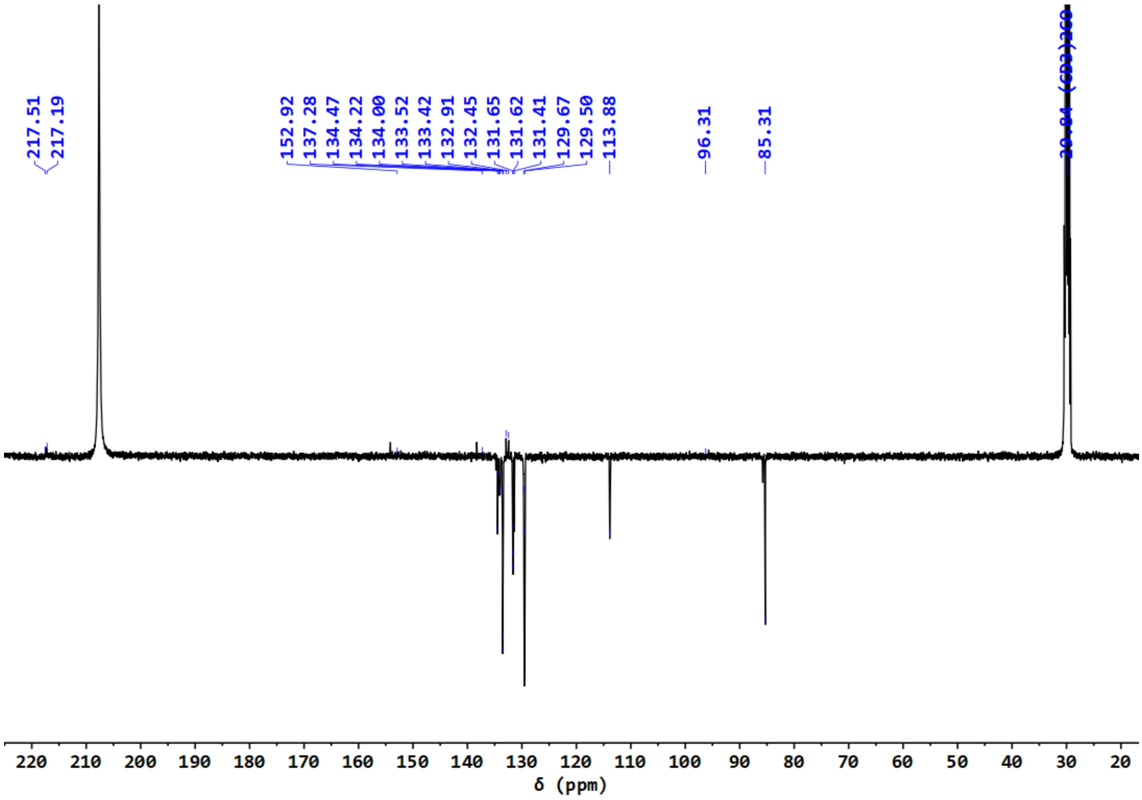

Figure S21 – $^{13}C\{^{1}H\}$-apt NMR spectrum of complex **4**, in acetone-d$_6$

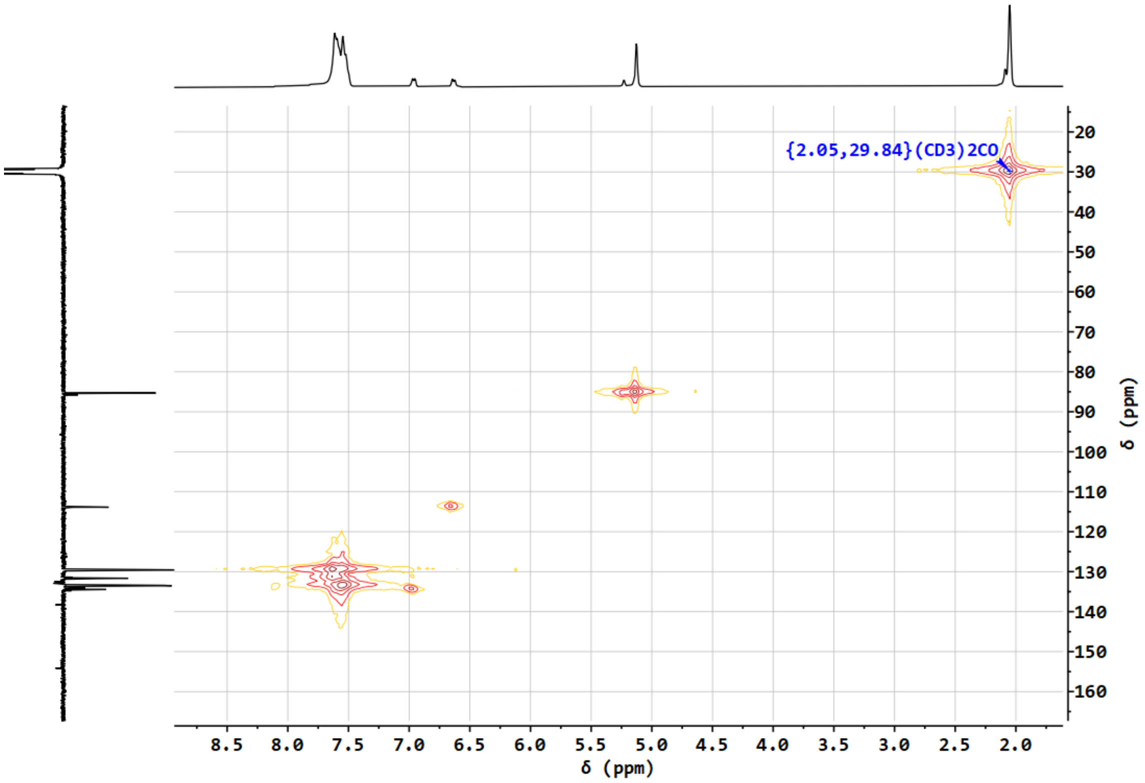

Figure S22 – HMQC spectrum of complex **4**, in acetone-d$_6$

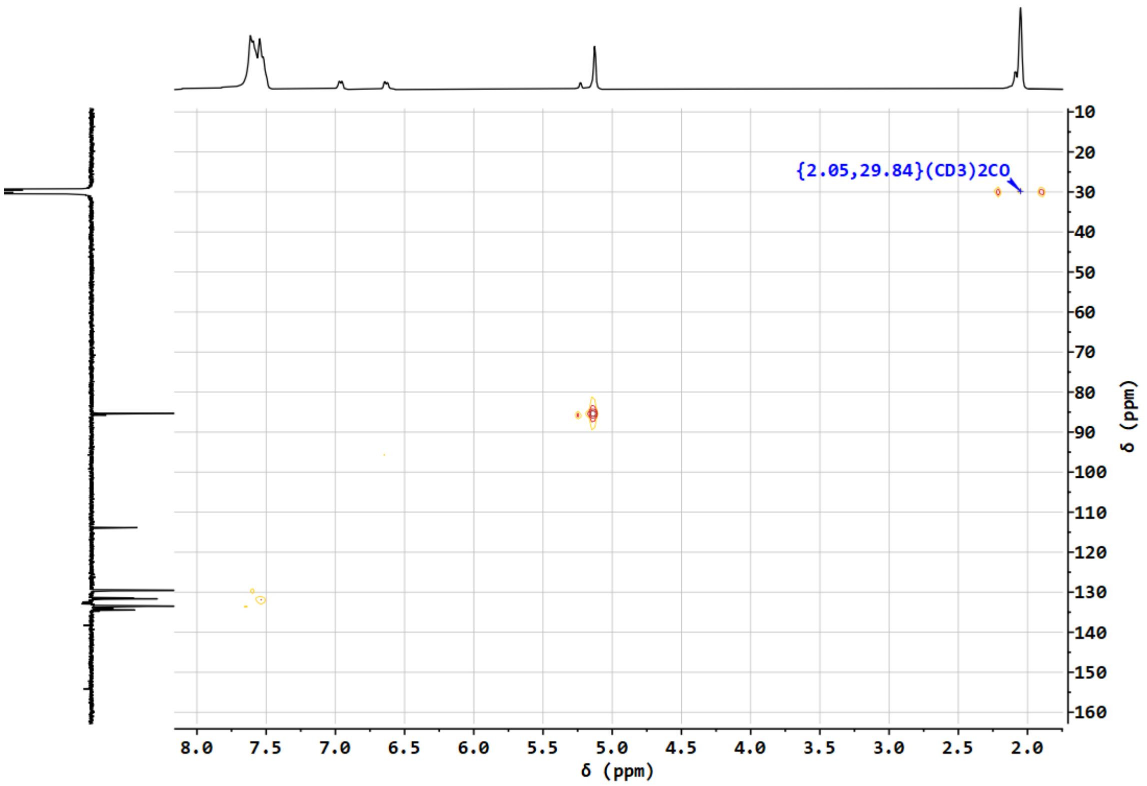

Figure S23 – HMBC spectrum of complex **4**, in acetone-d_6

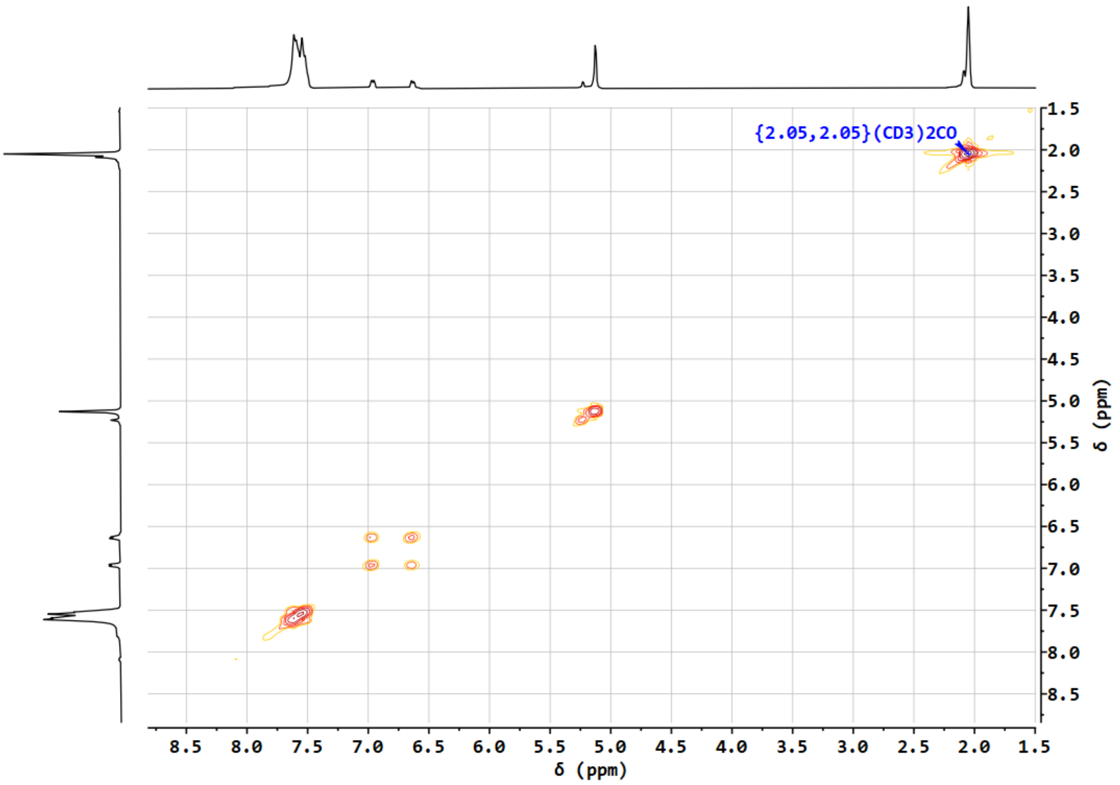

Figure S24 – COSY spectrum of complex **4**, in acetone-d_6

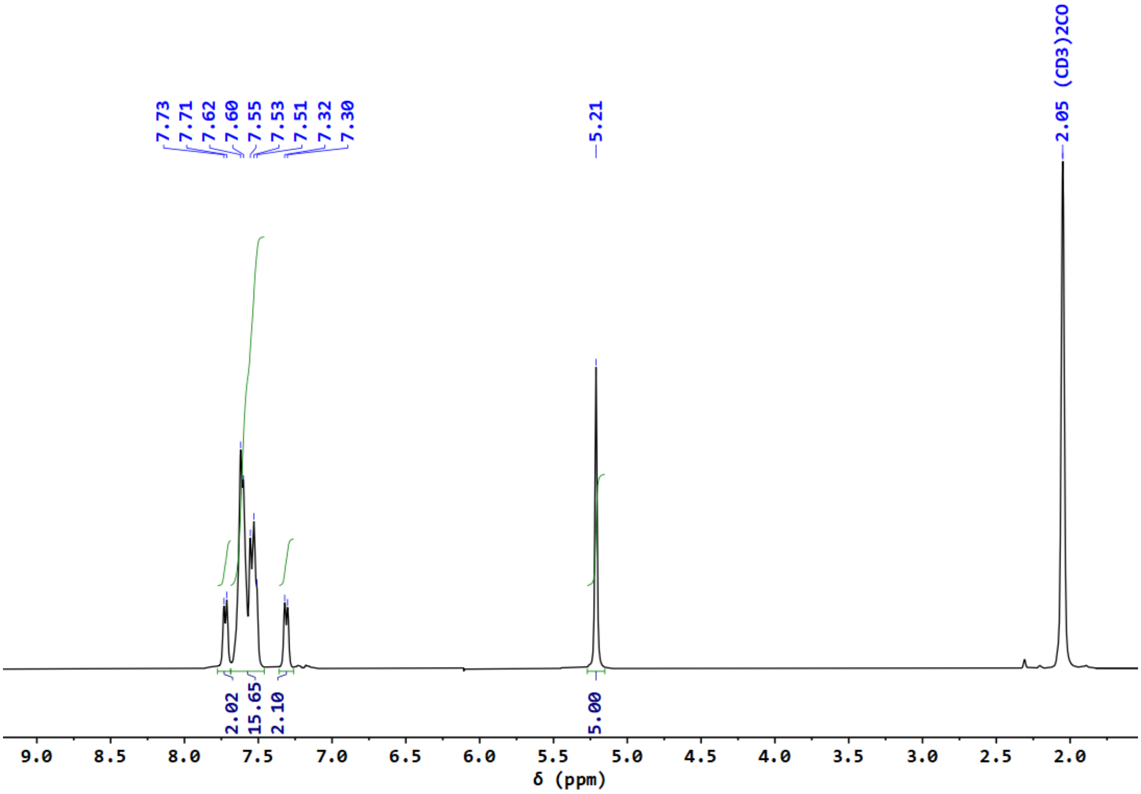

Figure S25 - ^{1}H NMR spectrum of complex **5**, in acetone-d_6

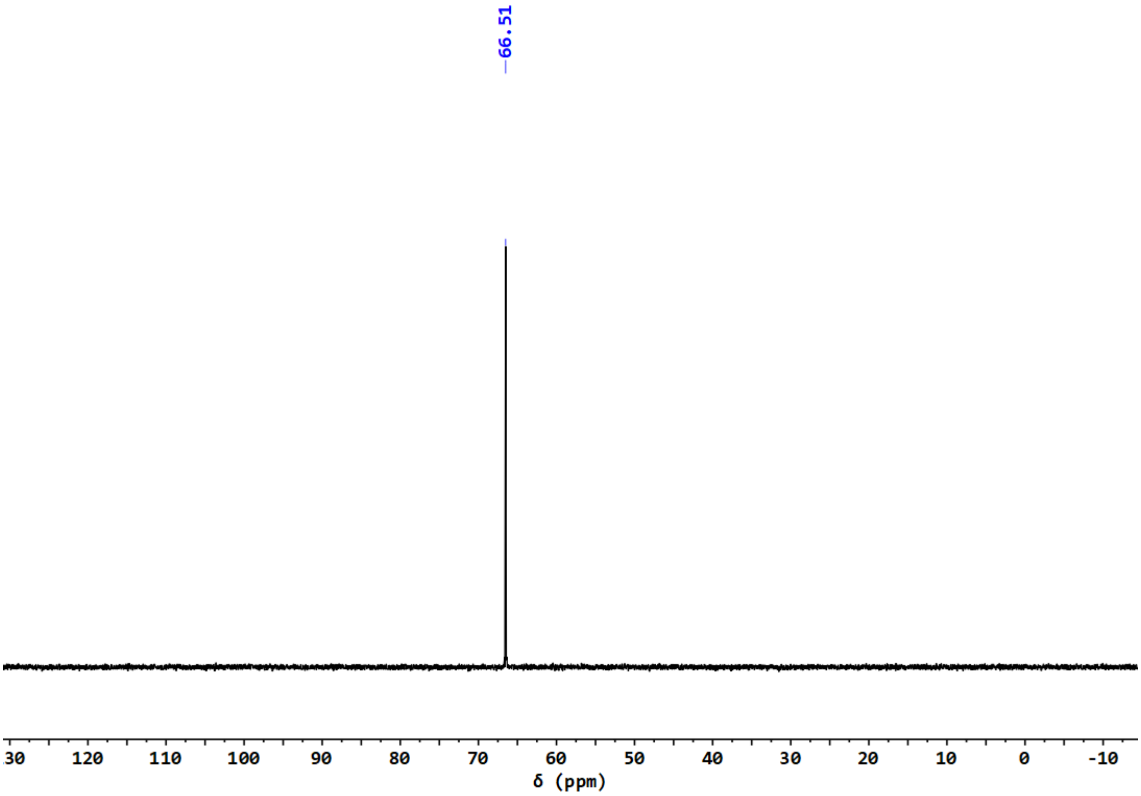

Figure S26 – $^{31}P\{^{1}H\}$ NMR spectrum of complex **5**, in acetone-d_6

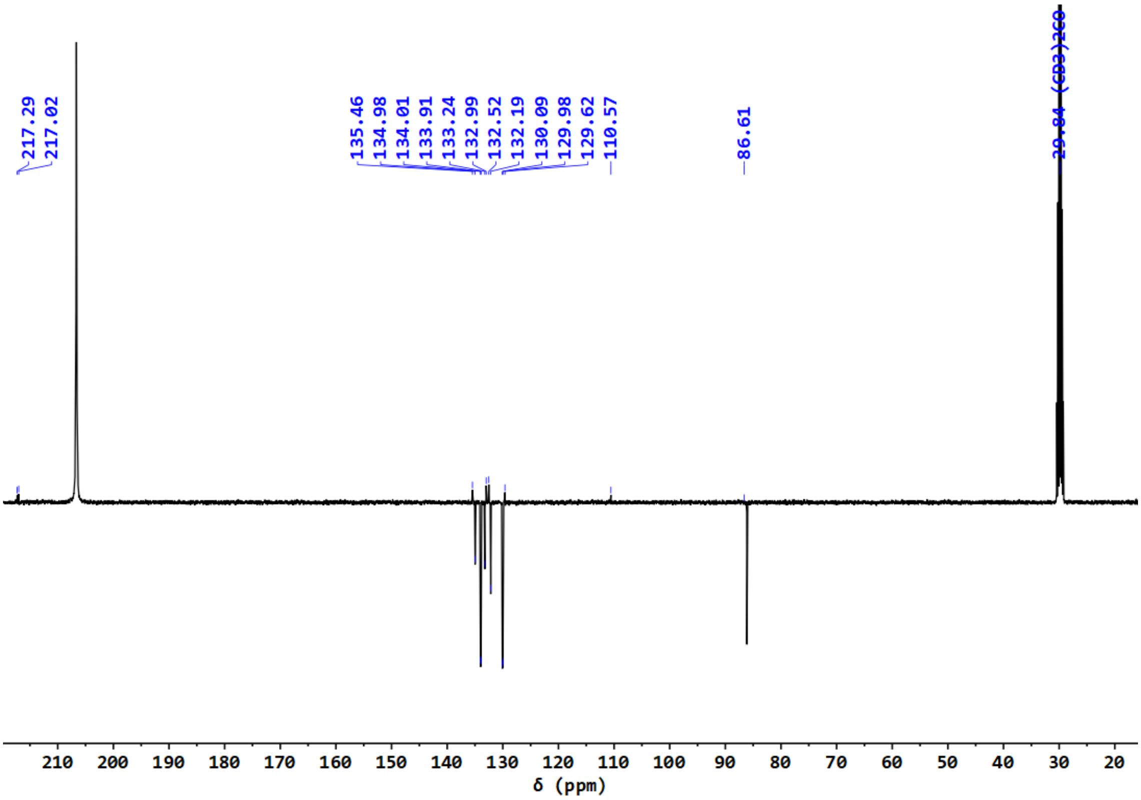

Figure S27 – $^{13}C\{^{1}H\}$-apt NMR spectrum of complex **5**, in acetone-d_6

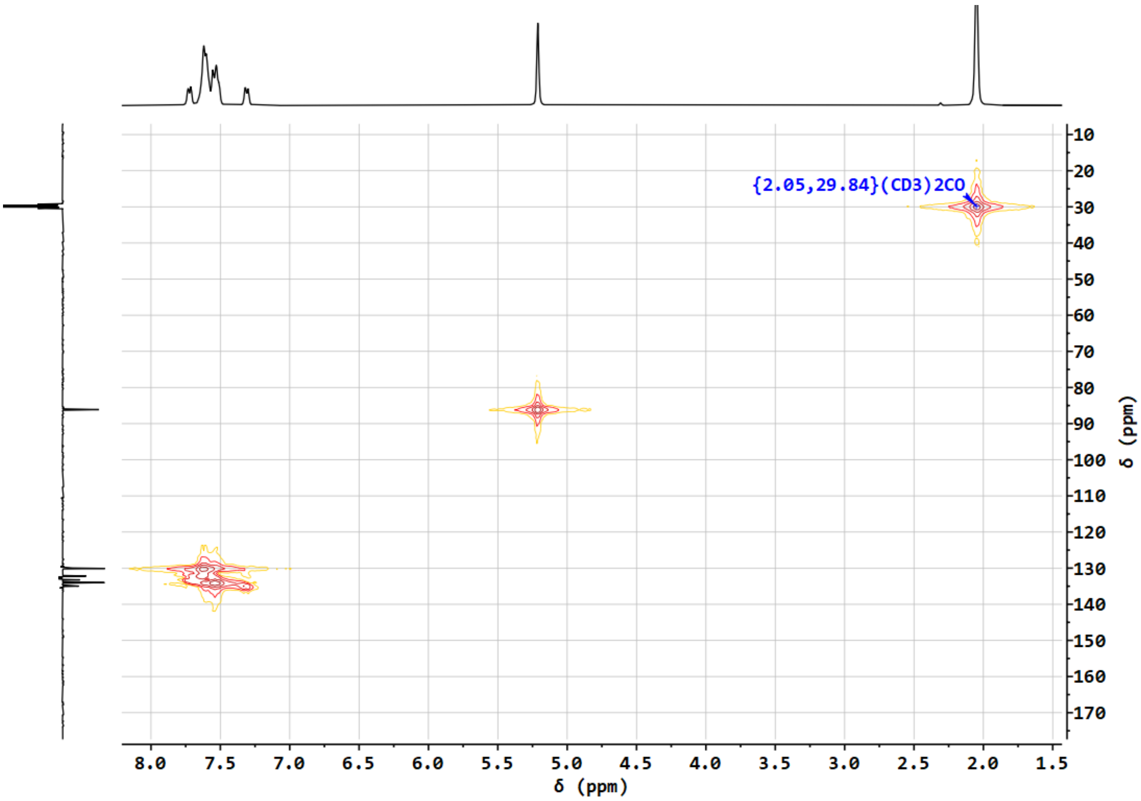

Figure S28 – HMQC spectrum of complex **5**, in acetone-d_6

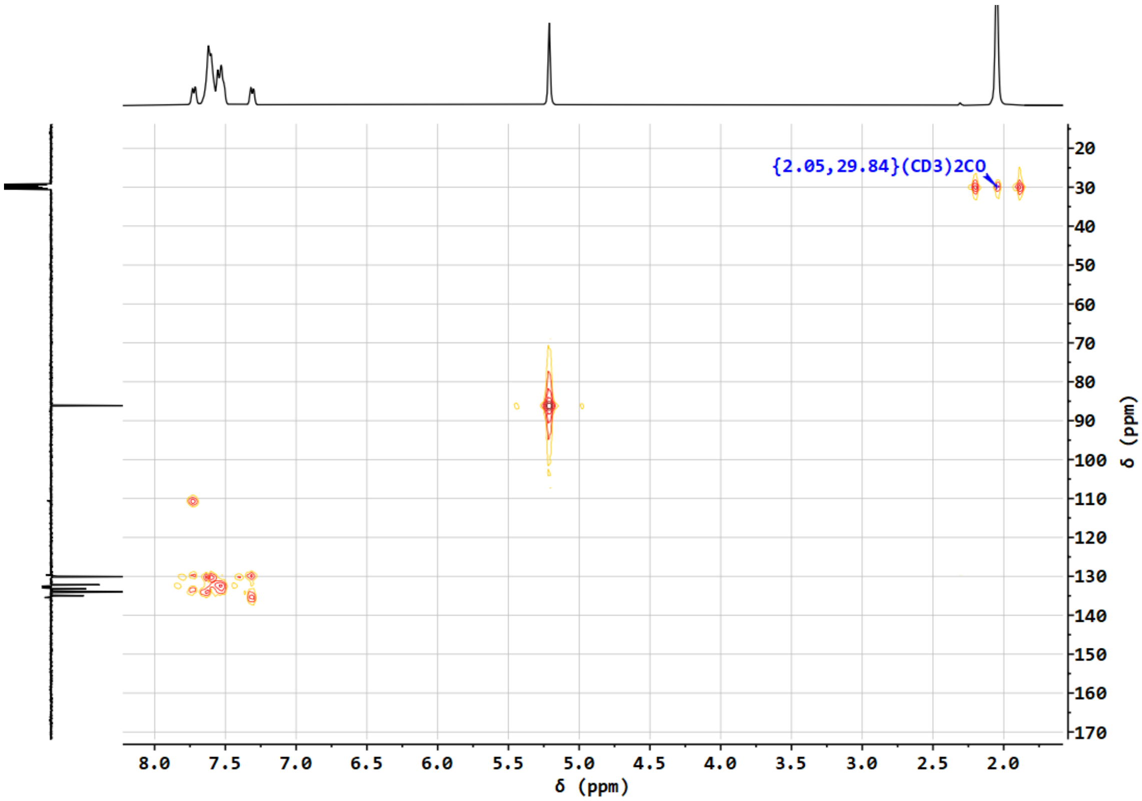

Figure S29 – HMBC spectrum of complex **5**, in acetone-d_6

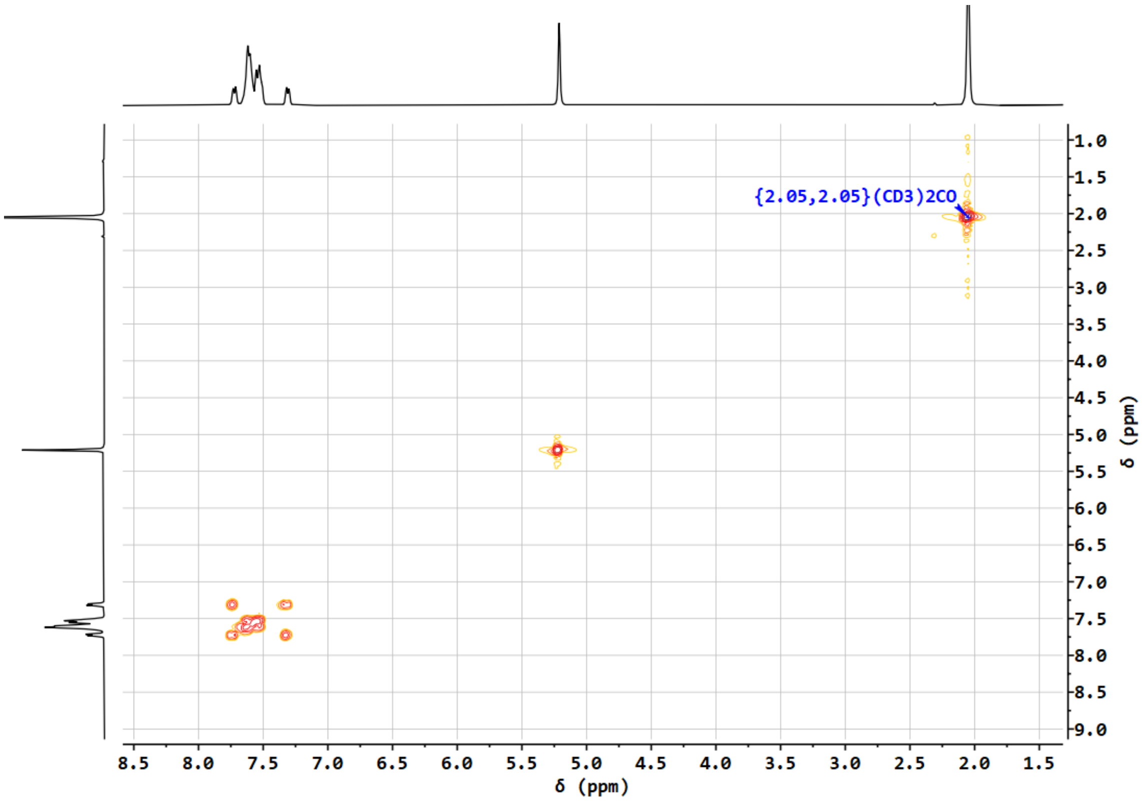

Figure S30 – COSY spectrum of complex **5**, in acetone-d_6

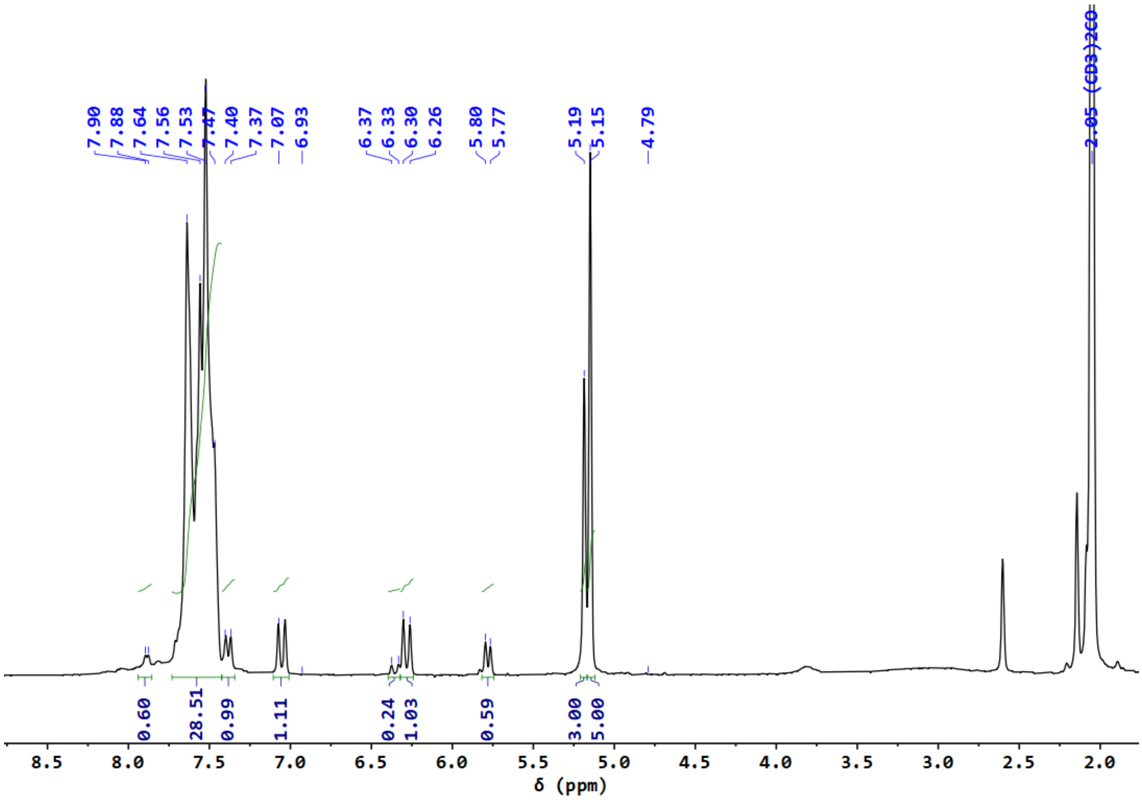

Figure S31 - ^{1}H NMR spectrum of complex **6**, in acetone-d_6

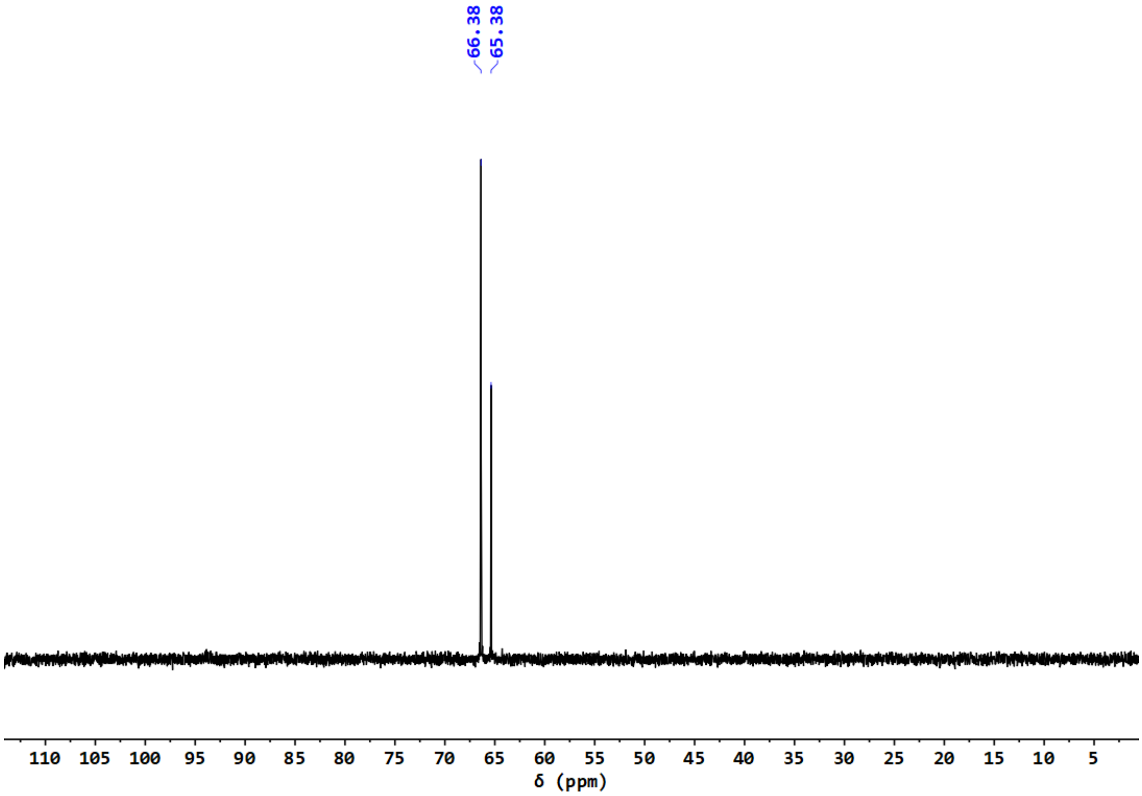

Figure S32 – ^{31}P{^{1}H} NMR spectrum of complex **6**, in acetone-d_6

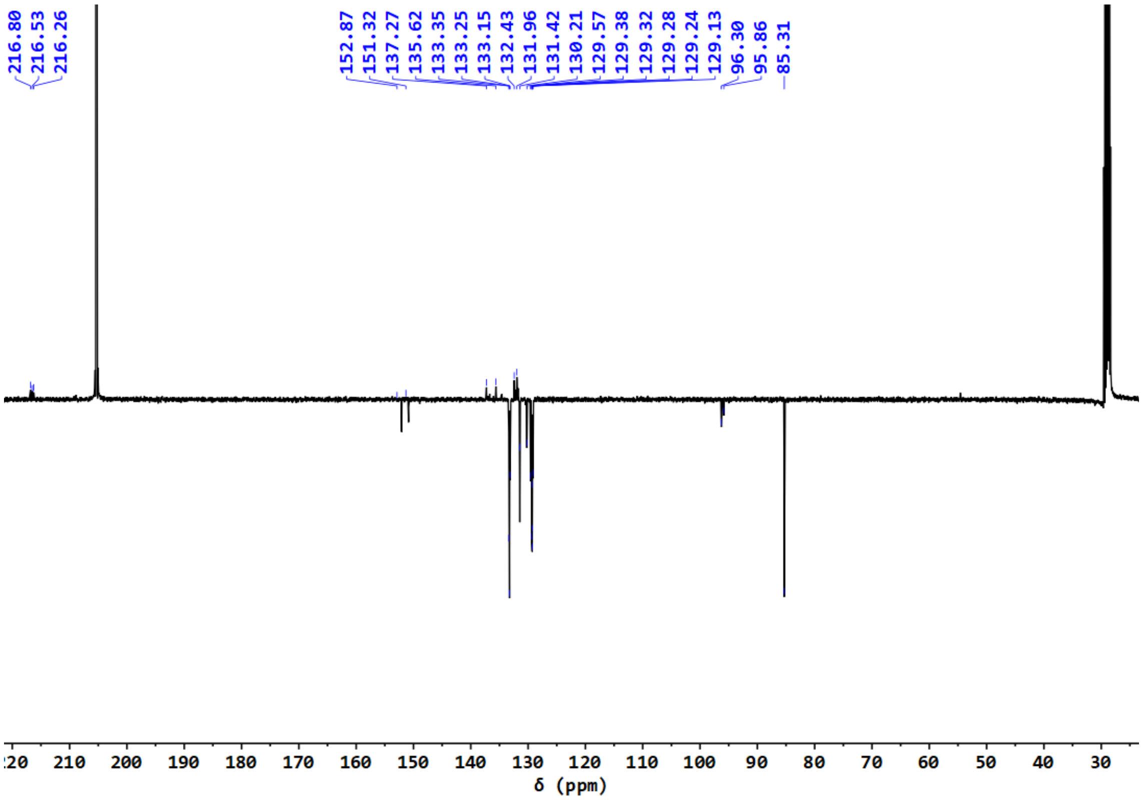

Figure S33 – $^{13}C\{^{1}H\}$-apt NMR spectrum of complex **6**, in acetone-d_6

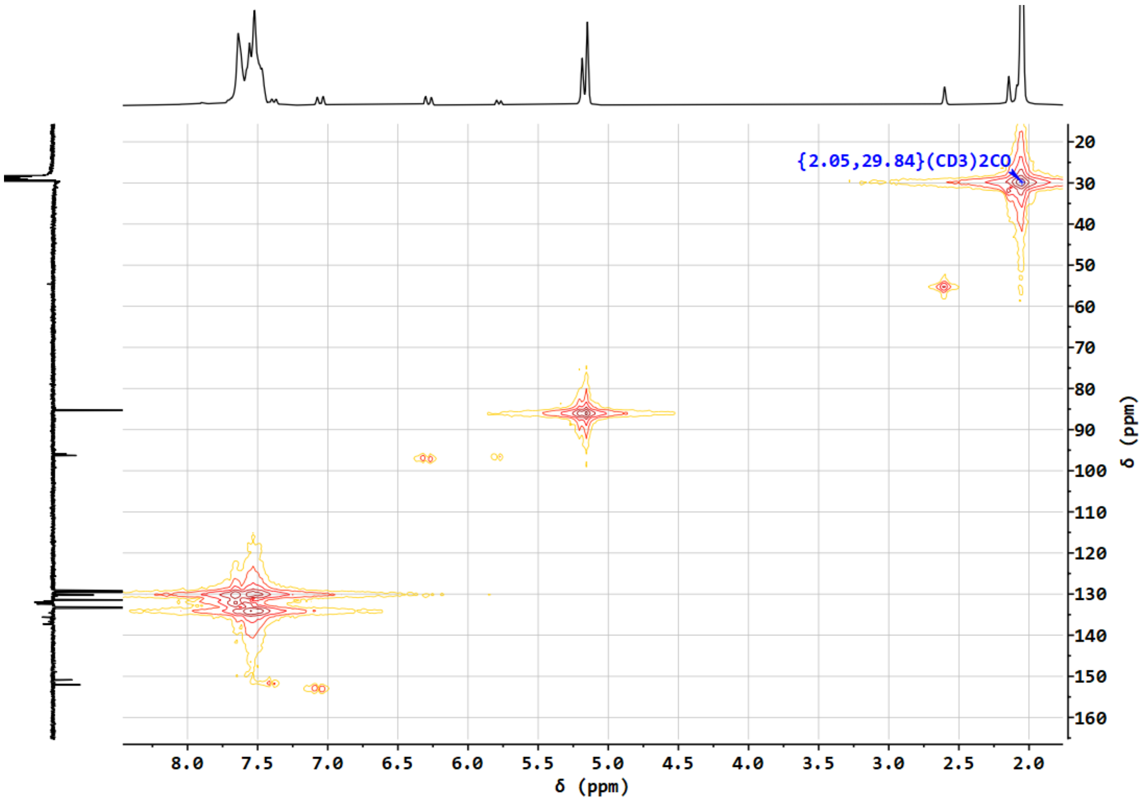

Figure S34 – HMQC spectrum of complex **6**, in acetone-d_6

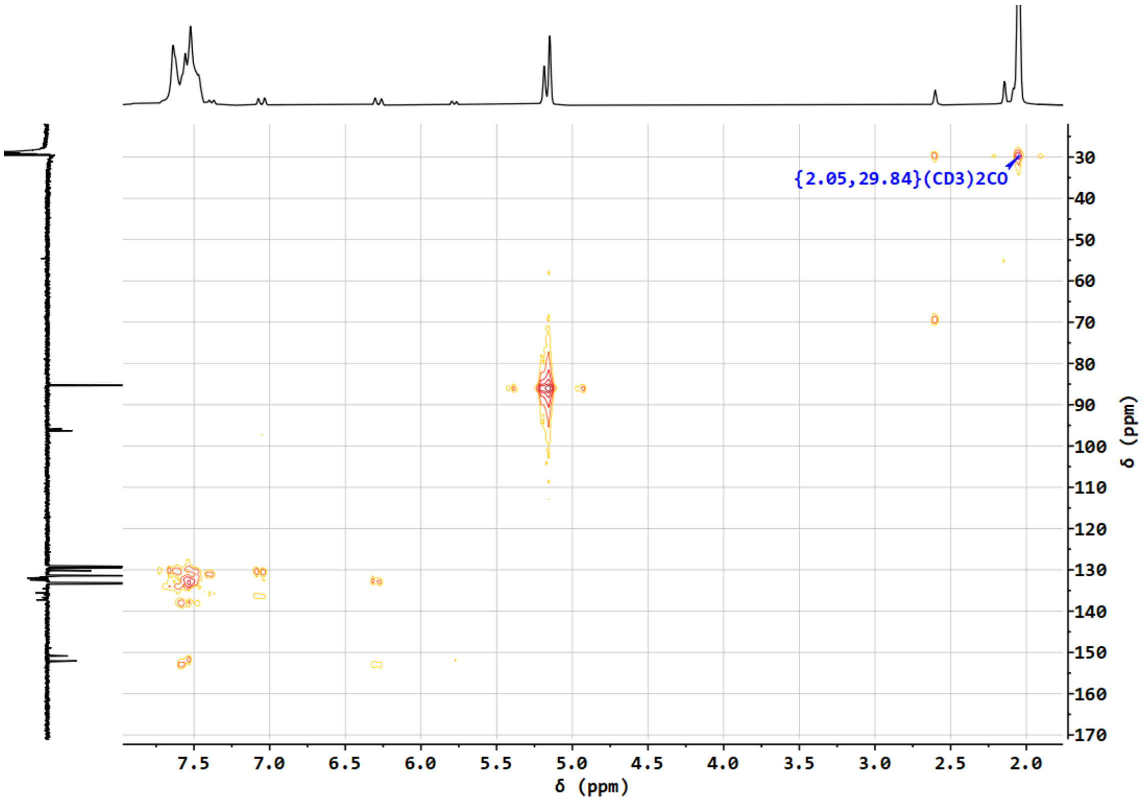

Figure S35 – HMBC spectrum of complex **6**, in acetone-d_6

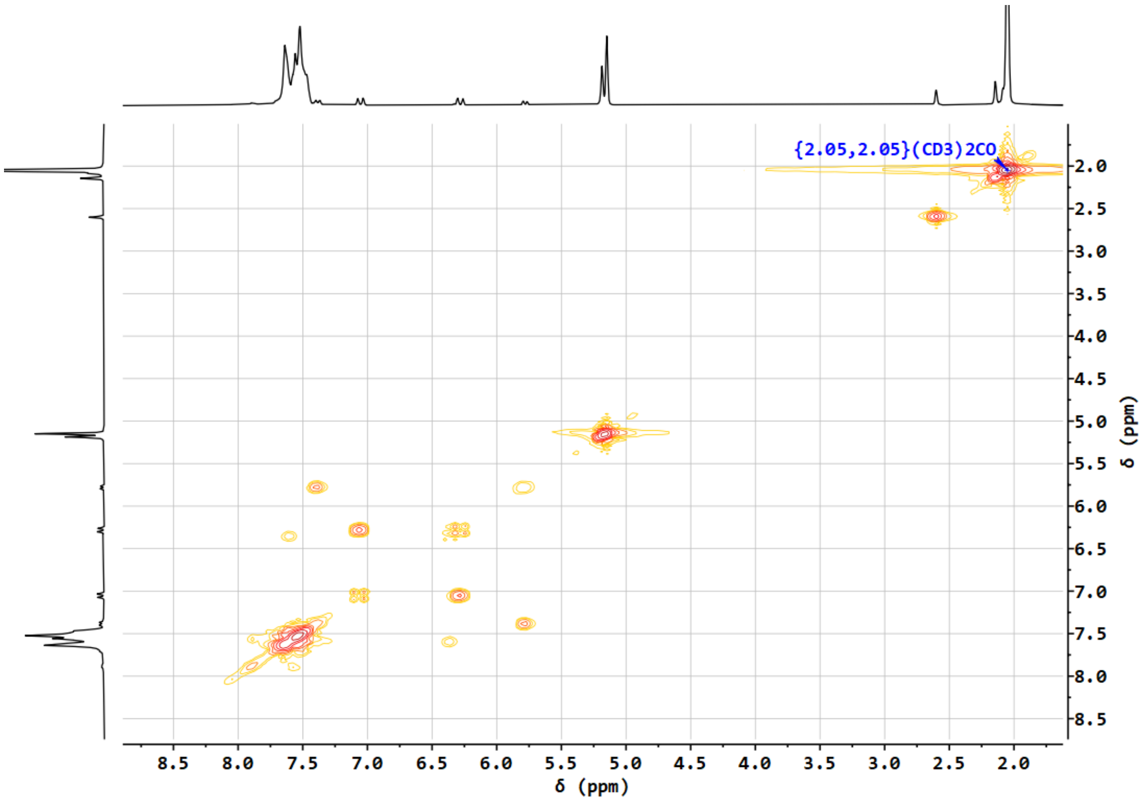

Figure S36 – COSY spectrum of complex **6**, in acetone-d_6

Table S1. Bond lengths [Å] and angles [°] for [Fe(η[5]-Cp)(CO)(PhCN)(PPh$_3$)][CF$_3$SO$_3$] **1**, [Fe(η[5]-Cp)(CO)(*p*-NCPhNH$_2$)(PPh$_3$)][CF$_3$SO$_3$] **4** and [Fe(η[5]-Cp)(CO)(*p*-NCPhBr)(PPh$_3$)][CF$_3$SO$_3$] **5**.

Bond lengths	**1**	**4**	**5**
Fe(1)-C(1M)	1.772(3)	1.7692(19)	1.799(4)
Fe(1)-C(1)	2.121(3)	2.101(2)	2.112(4)
Fe(1)-C(2)	2.099(3)	2.0726(19)	2.115(4)
Fe(1)-C(3)	2.079(3)	2.082(2)	2.097(4)
Fe(1)-C(4)	2.076(3)	2.102(2)	2.080(4)
Fe(1)-C(5)	2.097(3)	2.122(2)	2.093(4)
Fe(1)-N(1)	1.905(3)	1.9101(16)	1.917(3)
Fe(1)-P(1)	2.2314(9)	2.2224(6)	2.2598(9)
O(1M)-C(1M)	1.152(4)	1.144(2)	1.134(4)

Bond angles	**1**	**4**	**5**
C(1M)-Fe(1)-N(1)	97.42(12)	97.73(8)	97.88(14)
C(1M)-Fe(1)-C(4)	89.35(14)	155.63(8)	95.87(18)
N(1)-Fe(1)-C(4)	150.18(12)	101.41(7)	157.33(15)
C(1M)-Fe(1)-C(3)	121.40(14)	121.44(8)	135.79(17)
N(1)-Fe(1)-C(3)	140.97(12)	140.51(7)	125.78(15)
C(4)-Fe(1)-C(3)	39.82(13)	39.65(8)	40.76(18)
C(1M)-Fe(1)-C(5)	93.98(14)	128.62(9)	83.94(17)
N(1)-Fe(1)-C(5)	110.55(12)	86.71(8)	123.69(14)
C(4)-Fe(1)-C(5)	39.76(13)	38.98(9)	40.40(16)
C(3)-Fe(1)-C(5)	66.21(13)	65.79(8)	67.25(16)
C(1M)-Fe(1)-C(2)	155.92(14)	89.40(8)	149.59(17)
N(1)-Fe(1)-C(2)	102.14(12)	149.26(8)	92.76(14)
C(4)-Fe(1)-C(2)	66.75(14)	66.51(8)	66.76(17)
C(3)-Fe(1)-C(2)	39.44(12)	39.67(8)	38.41(16)
C(5)-Fe(1)-C(2)	66.10(14)	65.98(8)	66.57(16)
C(1M)-Fe(1)-C(1)	128.98(14)	93.53(9)	111.15(17)
N(1)-Fe(1)-C(1)	87.76(12)	109.40(8)	91.49(14)
C(4)-Fe(1)-C(1)	65.93(14)	65.96(9)	66.69(16)
C(3)-Fe(1)-C(1)	65.63(13)	66.54(9)	65.95(15)
C(5)-Fe(1)-C(1)	38.71(13)	38.79(8)	39.07(15)
C(2)-Fe(1)-C(1)	39.32(13)	40.06(8)	39.85(15)
C(1M)-Fe(1)-P(1)	90.40(10)	90.90(6)	95.75(13)
N(1)-Fe(1)-P(1)	93.36(8)	93.52(5)	89.20(9)
C(4)-Fe(1)-P(1)	115.70(10)	102.76(6)	107.27(12)
C(3)-Fe(1)-P(1)	90.45(9)	90.94(6)	91.81(11)
C(5)-Fe(1)-P(1)	154.85(10)	140.14(7)	146.92(11)
C(2)-Fe(1)-P(1)	102.23(10)	116.34(6)	112.87(12)
C(1)-Fe(1)-P(1)	140.16(10)	155.80(7)	152.72(11)

Table S2. Relevant TD-DFT (PBE0) excitation energies (λ), oscillator strengths (f) and compositions (only those > 5% are shown), for complexes **1**-**6**, compared with experimental data (λ_{exp}). Both calculated and experimental values were obtained in dichloromethane.

**Complex**	**λ / nm**	***f***	**Composition**	**λ_{exp} / nm** (ε/M $^{-1}$ cm $^{-1}$)
**1**	515	0.0028	H → L+1 (28%)	502 (225)
			H → L+2 (13%)	
			H → L+4 (9%)	
			H-1 → L+1 (7%)	
	397	0.0177	H → L+4 (24%)	390 (990)
			H → L+1 (14%)	
			H-5 → L+1 (10%)	
			H-2 → L+1 (6%)	
			H → L+2 (5%)	
**2**	520	0.0029	H → L+1 (25%)	546 (103)
			H → L+2 (13%)	507 (143)
			H → L+4 (9%)	
			H-10 → L+1 (6%)	
			H → L (5%)	
	398	0.0175	H → L+4 (16%)	407 (492)
			H-3 → L+1 (10%)	
			H → L+1 (10%)	
			H-1 → L+4 (9%)	
**3**	516	0.0028	H → L+1 (28%)	621 (65)
			H → L+2 (13%)	546 (217)
			H → L+4 (10%)	
			H-1 → L+1 (8%)	
	398	0.0177	H → L+1 (14%)	397 (1290)
			H → L+4 (24%)	
			H-3 → L+1 (9%)	
			H → L+2 (5%)	
**4**	525	0.003	H-1 → L (22%)	525 (440)
			H-1 → L+4 (10%)	
			H-1 → L+2 (9%)	
			H → L (7%)	
			H-10 → L (5%)	
			H-2 → L (5%)	
	400	0.0175	H-1 → L+4 (25%)	406 (1245)
			H-3 → L (10%)	
			H-1 → L (12%)	
**5**	515	0.0028	H → L+1 (30%)	515 (323)
			H → L+2 (15%)	
			H-1 → L+1 (7%)	
			H-9 → L+1 (6%)	
			H → L+4 (5%)	

	397	0.0175	H → L+5 (16%)<br>H → L+1 (12%)<br>H-3 → L+1 (10%)<br>H → L+2 (6%)<br>H → L+4 (6%)<br>H-4 → L+1 (5%)	404 (1337)
**6**	525	0.0029	H → L+1 (28%)<br>H → L+2 (13%)<br>H-10 → L+1 (5%)<br>H-2 → L+1 (5%)	532 (408)
	404	0.032	H → L+1 (13%)<br>H → L+4 (14%)<br>H-3 → L+1 (7%)<br>H → L+3 (7%)<br>H → L+2 (5%)	409 (1233)

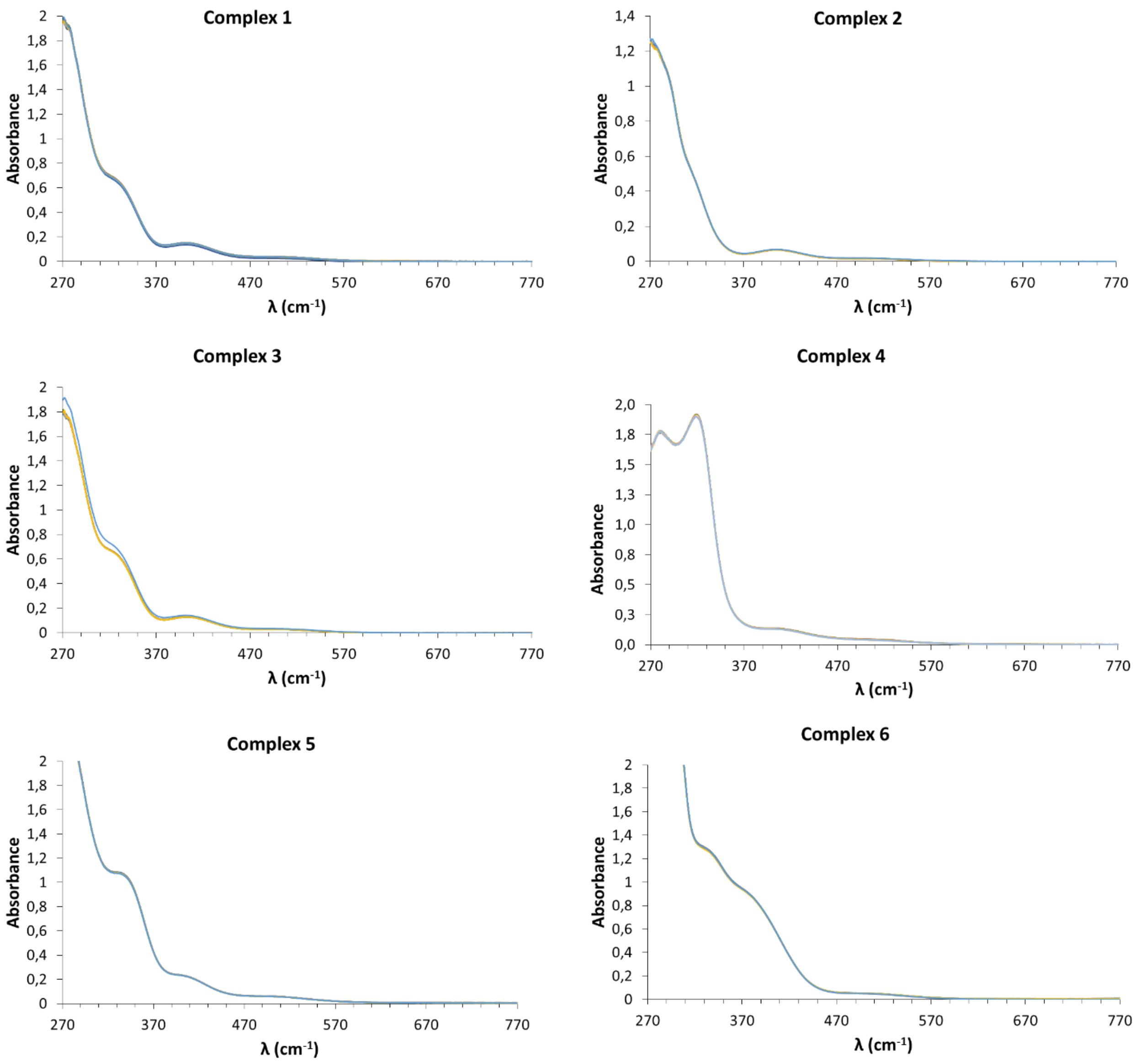

Figure S37 - UV-Vis spectra of complexes **1 - 6** in DMSO along the 24 h study.

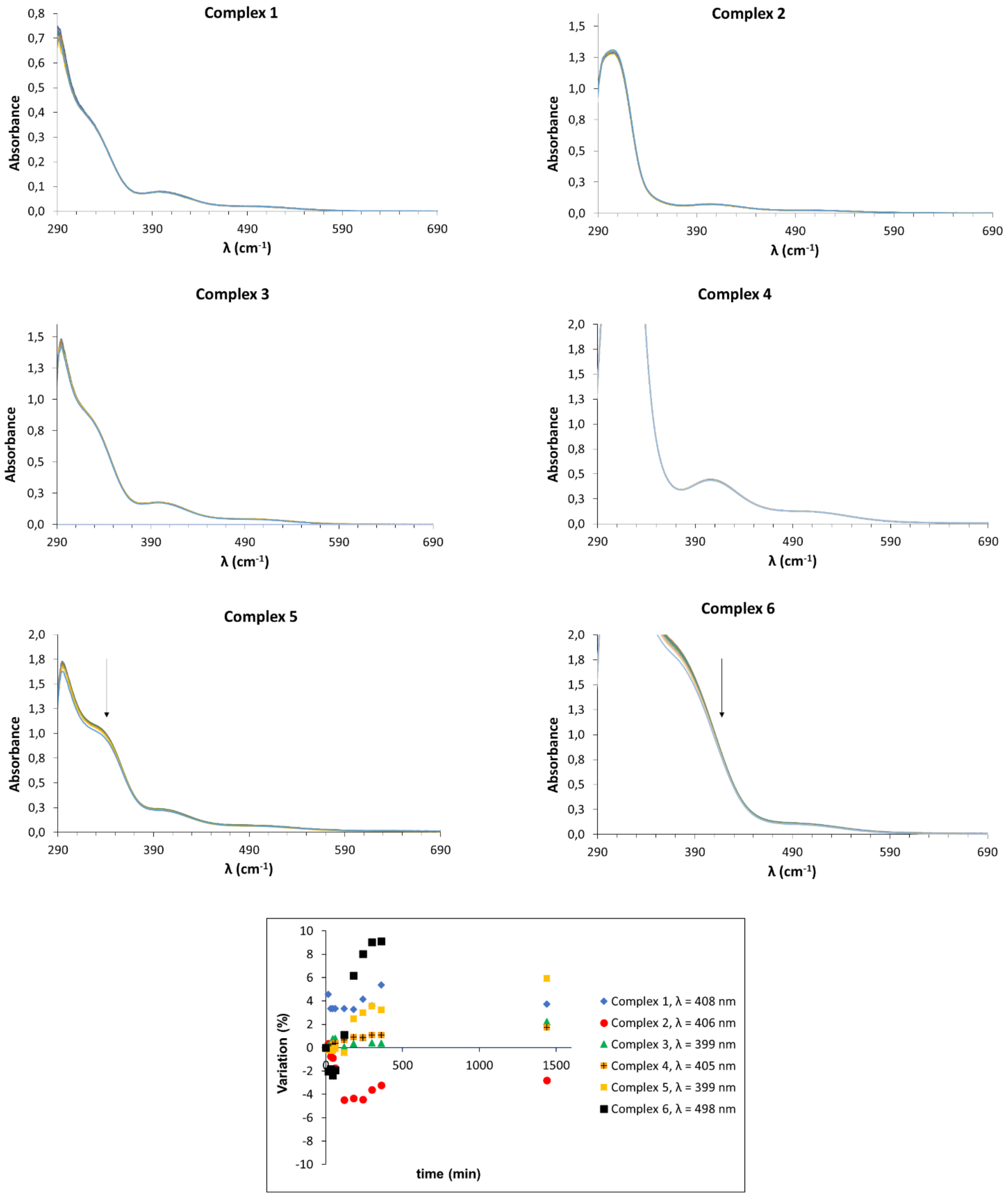

Figure S38 - UV-Vis spectra of complexes **1** - **6** in DMSO/DMEM mixture along the 24 h study and its variation plot (%) (bottom).

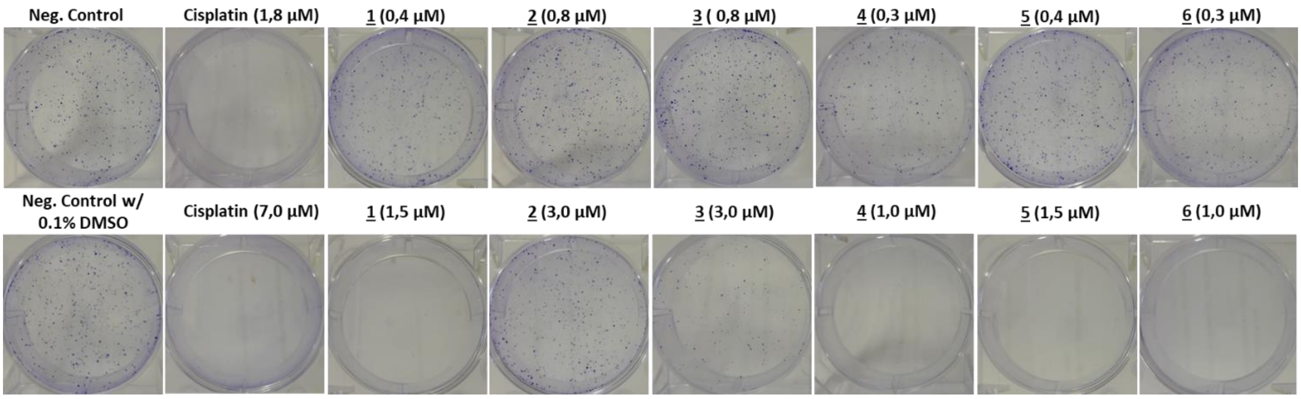

Figure S39. ‘FeCp’ compounds affect the colony formation ability of SW480 cell line. Analysis of the colony formation ability, after 48 h of incubation with 1/4 IC_{50} and IC_{50}, in SW480 cell line. Representative images of colony formation assay in SW480 cell line.

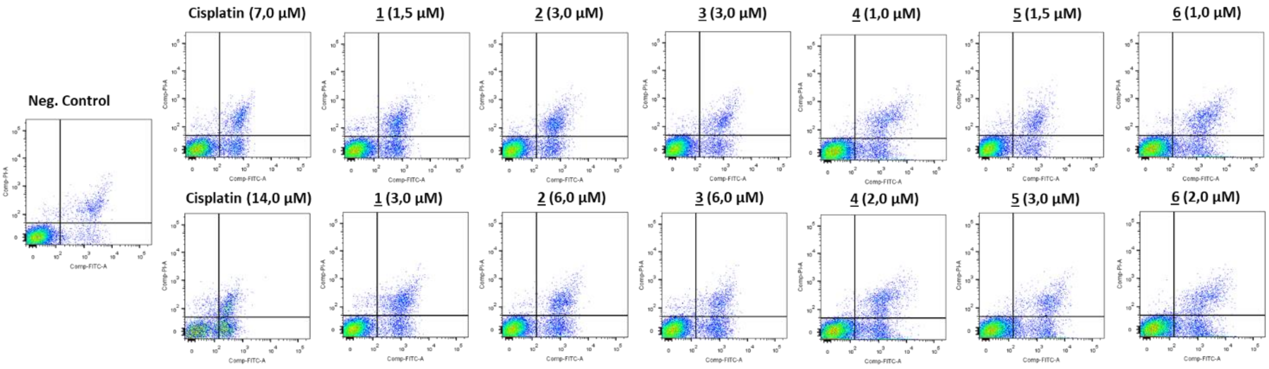

Figure S40. ‘FeCp’ compounds induce apoptosis in SW480 colorectal cancer-derived cell line. Apoptotic cell death was analyzed by Annexin V fluorescein isothiocyanate (AV-FITC) and propidium iodide (PI) assay in SW480 cells, after incubation with IC_{50} and $2\times IC_{50}$ concentrations for 48 h. Representative histograms of SW480 cell line double stained with AV and PI.

Table S4. Crystal data and structure refinement for [Fe(η^5-Cp)(CO)(PhCN)(PPh_3)][CF_3SO_3] **1**, [Fe(η^5-Cp)(CO)(p-NCPhNH_2)(PPh_3)][CF_3SO_3] **4** and [Fe(η^5-Cp)(CO)(p-NCPhBr)(PPh_3)][CF_3SO_3] **5**.

	**1**	**4**	**5**
Formula	$C_{32}H_{25}F_3FeNO_4PS$	$C_{32}H_{26}F_3FeN_2O_4PS$	$C_{32}H_{24}BrF_3FeNO_4PS$
Formula weight	663.41	678.43	742.31
T, *K*	100(2)	100(2)	100(2)
Wavelength, Å	0.71073	0.71073	0.71073
Crystal system	Monoclinic	Monoclinic	Monoclinic
Space group	$P2_1/c$	$P2_1/c$	$P2_1/c$
a/Å	12.0421(5)	11.9877(12)	10.5753(9)
b/Å	20.0232(10)	20.0784(15)	14.2460(11)
c/Å	11.9383(5)	12.1720(11)	19.8010(16)
β/°	93.619(3)	94.652(4)	93.620(3)
V/Å^3	2872.8(2)	2920.1(5)	2977.2(4)
Z	4	4	4
F_{000}	1360	1392	1496
D_{calc}/g cm^{-3}	1.534	1.543	1.656
μ/mm^{-1}	0.713	0.704	2.031
θ/ (°)	1.99 to 26.40	1.96 to 28.35	1.93 to 28.28
R_{int}	0.1090	0.0742	0.0958
Crystal size/ mm^3	0.23 x 0.14 x 0.07	0.15 x 0.10 x 0.04	0.25 x 0.04 x 0.03
Goodness-of-fit on F^2	1.042	1.033	1.049
R_1 [a]	0.0457	0.0345	0.0470
wR_2 (all data) [b]	0.1030	0.0801	0.1064
Largest differences peak and hole (eÅ^{-3})	0.631 and -0.461	0.378 and -0.365	1.279 and -0.543

[a] $R_1 = \Sigma||F_o|-|F_c||/\Sigma|F_o|$. [b] $wR_2 = \{\Sigma[w(||F_o|^2-|F_c|^2|)^2]|/\Sigma[w(F_o^2)^2]\}^{1/2}$
